# Cancer-associated SF3B1 mutation suppresses DNA repair by disrupting the organization of nuclear actin network

**DOI:** 10.1038/s41419-026-08569-5

**Published:** 2026-03-21

**Authors:** Rui Qian, Zhipeng Zhao, Xuanxuan Sun, Benkai Xin, Peipei An, Ting Yang, Ning Wu, Xin Hu, Youzhong Wan

**Affiliations:** 1 Cancer Biology Laboratory, China-Japan Union Hospital of Jilin University, Jilin University, Changchun, China; 2 Jilin Provincial Key Laboratory of Cancer Biology, Changchun, China; 3 Department of Radiation Oncology, China-Japan Union Hospital of Jilin University, Jilin University, Changchun, China; 4 Department of Cardiology, China-Japan Union Hospital of Jilin University, Jilin University, Changchun, China

**Keywords:** Small RNAs, Double-strand DNA breaks, Oncogenes

## Abstract

Nuclear actin filament is required for efficient repair of DNA double-strand breaks. While cancer-associated SF3B1 mutation leads to impaired DNA repair, the underlying mechanism remains elusive. Here, we found that SF3B1 mutation led to defective nuclear actin network during DNA repair. Mechanistically, SF3B1 mutation increased the expression of circATP9B, which interacted with and facilitated the degradation of MYH9. MYH9 deficiency abolished the assembly of nuclear actin network, which, in turn, suppressed the movement and clustering of DNA damage foci, resulting in inefficient DNA repair. Together, our study reveals a novel mechanism by which SF3B1 mutation influences cancer progression *via* circRNA, and underscores the important role of MYH9 in organization of nuclear actin network.

## Introduction

Spliceosome factor 3b subunit 1(SF3B1) is frequently mutated in a variety of cancer, including myelodysplastic syndrome (MDS) ( ~ 20%) [1, 2], chronic lymphoid leukemia ( ~ 15%) [3], breast cancer ( ~ 2%) [4], pancreatic cancer ( ~ 2%) [5, 6], etc. SF3B1 mutations in cancer are usually heterozygous mutations at several hotspots, and the most common SF3B1 mutation is an A to G transition that results in lysine to glutamic acid substitution at amino acid position 700 (SF3B1- K700E). SF3B1 is an essential component of U2 snRNP in RNA splicing machinery, and cancer-associated SF3B1 mutation (hereafter, SF3B1 mutation) causes aberrant RNA splicing, which leads to degradation of the aberrantly spliced mRNA through non-sense mediated decay (NMD) or changed codon [7]. Multiple such aberrantly spliced mRNA has been demonstrated to promote cancer progression [8–10]. In addition, SF3B1 mutation destabilizes genomic DNA via stalled replication forks and increased R-loop formation [11–13]. SF3B1 mutation also disrupts DNA repair, especially homology-directed repair (HDR) of double-strand breaks (DSBs), thereby enhancing the sensitivity of cancer cells to PARP inhibitors through synthetic lethality [14, 15]. Notably, SF3B1 mutation leads to accumulation of unrepaired DSBs at late time points following DNA damage infliction, suggesting a deficiency in the repair of DSBs that are typically resolved at later stages of DNA repair [15]. However, the mechanism responsible for such impaired DNA repair remains unclear.

HDR in heterochromatin domains requires the relocation of DSBs to nuclear periphery to prevent the aberrant recombination of highly repetitive DNA sequences at heterochromatin, and therefore DSBs at heterochromatin are usually resolved at later stages of DNA repair [16, 17]. Assembly of nuclear actin filaments is required for the relocation of DSBs in heterochromatin domains, and suppression of nuclear actin polymerization results in accumulation of unrepaired DNA damage foci at late time points following irradiation [18]. Moreover, nuclear actin polymerization drives the clustering of DSBs in both heterochromatin and euchromatin domains for efficient HDR [19]. Myosins are mechanoenzymes responsible for generating contractility and enabling the movement of cargo along actin filaments [20]. A deficiency in either MYO1A or MYO1B, two cargo-associated myosins, leads to defects in DSB trafficking along nuclear actin filaments [18].

Circular RNAs (circRNAs) are formed through back-splicing of pre-mRNA, a process that is dependent on canonical RNA spliceosome machinery [21, 22]. Multiple RNA splicing factors, including SF3B1, are involved in circRNA biogenesis [23, 24]. To investigate whether SF3B1 mutation can promote cancer progression through circRNA, we conducted circRNA sequencing in multiple cell lines with SF3B1-K700E mutation. We found that SF3B1 mutation led to an increase expression of circATP9B, which interacted with and facilitated the degradation of MYH9 (Non muscle myosin heavy chain IIA), a member of the myosin family essential for the organization of cellular actin filaments [25–27]. Importantly, MYH9 deficiency has been reported to result in severe DNA damage and promote progression of prostate cancer and squamous cell carcinoma [28, 29]. Therefore, we hypothesized that MYH9 plays a crucial role in the organization of nuclear actin filaments during DNA repair, and decreased MYH9 expression in cells with SF3B1 mutation leads to impaired DNA repair through destabilizing the networks of nuclear actin filaments, thus promoting cancer progression.

## Materials and methods

### In vivo Xenograft tumor models

All animals were maintained at Changchun Wish Technology Company and the experiments were performed using 5-week-old BALB/c female nude mice (Charles River Laboratories, China) under pathogen-free conditions. Each mouse received a single subcutaneous injection of Hela cells (1 × 10^7^/100 μL of a PBS and Matrigel suspension) stably expressing the circATP9B overexpression plasmid or the corresponding control vector. When the tumor volume reached 100 mm^3^, tumor-bearing mice were randomized to receive 50 mg/kg olaparib intraperitoneally 5 days per week or the same volume of PBS (*n* = 6 per group). The agents were administered 15 times in 21 days. Tumor volume was assessed three times weekly. Finally, the tumors were resected and weighed, and the tumor volumes were calculated using the formula tumor volume = length × width^2^ / 2.

### Cell lines and cell culture

Hela and K562 *SF3B1*^*K700E*^ isogenic cell line was obtained from Cyagen Biosciences (Suzhou, China) and Ubigene Biosciences (Guangzhou, China), respectively. Human Hela and HEK293T cells were cultured in Dulbecco’s modified Eagle’s medium supplemented with 10% fetal bovine serum and 1% penicillin/streptomycin at 37 °C with 5% CO_2_. K562 cells were grown in RPMI1640 medium supplemented with 10% fetal bovine serum and 1% penicillin/streptomycin at 37 °C with 5% CO_2_.

### CircRNA-seq data analysis

Total RNA was extracted from parental Hela and Hela CRISPR *SF3B1*^*K700E*^ cells or HEK293T cells transfected with SF3B1 expression constructs using TRIzol reagent (Invitrogen, USA). CircRNA-seq was performed by OUTDO BIOTECH (Shanghai, China). Briefly, rRNA was removed from each sample using the RiboMinus Human/Mouse Transcriptome Isolation Kit (Invitrogen, USA), followed by RNase R (Epicenter, USA) treatment and RNA-seq library construction. The libraries were deep-sequenced using an Illumina NovaSeq 6000 platform. Sequencing reads were aligned to the human reference genome (GRCh38.p14, Ensembl) using BWA-mem (version 0.7.17), and back-splice junction sites were identified using CIRI2 (version 2.0.6). The R package “edge R” (version 3.36.0) was used to identify differentially expressed circRNAs using |fold change | ≥ 2 and *p* < 0.05 as selection criteria. The circRNA-seq data has been deposited in the GEO database under the accession number GSE292492. MDS patients circRNA-seq data was obtained from GSE database (BioProject ID PRJNA896500).

### Ionizing radiation

Ionizing irradiation (X-rays) was administered using an Elekta Synergy linear accelerator system (Elekta, Sweden) at a dose rate of 6 Gy/min for doses of 4 Gy.

### Plasmid construction, siRNA transfection, and lentiviral infection

For circATP9B expression constructs, a 472 bp DNA fragment containing circATP9B cDNA was cloned into the pLC5-ciR or pLO5-ciR vector (GENESEED, China) between the EcoRI and BamHI restriction sites. Human *MYH9*, *RAD52*, and *53BP1* were cloned into the pEZ-Lv151, pEZ-Lv197-mCherry, and pEZ-Lv197-EYFP vectors (GeneCopoeia, USA), respectively. Furthermore, Flag and NLS tags were inserted into the N-terminus of the pEZ-Lv151-MYH9 plasmid. Flag-MYH9 ΔTail domain (Δ778-1960 aa), Flag-MYH9 ΔHead domain (Δ78-777 aa), Flag-MYH9 ΔN terminal domain (Δ27-77 aa), pLO5-ciR-circATP9B Δ272-323 nt, pLO5-ciR-circATP9B Δ47-98 nt, pLO5-ciR-circATP9BΔ126-177 nt plasmids were synthesized by GenScript (Zhenjiang, China). The pnAC-Tag GFP (nuclear-actin-CB) plasmid was purchased from Chromotek. The SF3B1 expression constructs were synthesized and cloned as previously described [9]. For circATP9B knockdown, cells were transfected with siRNAs targeting circATP9B (GENESEED, China). The siRNAs targeting MYH9, MYO1A, and MYO1B were synthesized by Gene Pharma (Suzhou, China). The sequences for siRNAs are listed in Table S10.

Plasmid DNAs were transfected using Lipofectamine 2000 transfection reagents while siRNAs were transfected employing Lipofectamine RNAiMAX (Invitrogen, USA), both according to the manufacturer’s instructions. Transfected cells were harvested 48 h after transfection for further experiments. Lentiviral particles were produced *via* transfection of packaging vector psPAX2, envelope plasmid pMD2.G, and objective plasmid into HEK293T cells, and conditioned media containing lentiviral particles were harvested 48 h after transfection. Hela cells were infected with viral particles in the presence of 10 μg/mL Polybrene (Solarbio, China), and infection was confirmed by qRT-PCR or western blot.

### PCR and quantitative RT-PCR

Total RNA was isolated using *TransZol* Up reagent (Transgen Biotech, China). Genomic DNA was extracted from cells using a Genomic DNA kit (Transgen Biotech, China). For circRNA, reverse transcription was performed with random hexamers using an RT reagent Kit (Takara, China). For mRNA, reverse transcription was carried out using All-in-One First-Strand cDNA Synthesis SuperMix Kit (Transgen Biotech, China). PCR was performed using PrimeSTAR Max DNA Polymerase (Takara, China). PCR products were detected using 1% agarose gel electrophoresis and gel extraction was carried out using a TIANgel Midi Purification Kit (TIANGEN, China). qPCR was performed using One-Step qRT-PCR SuperMix (Transgen Biotech, China) in a LightCycler System (Roche, Switzerland). Relative RNA expression levels were calculated using the 2^-ΔΔCt^ method. The primers for qRT-qPCR, PCR and Taqman qPCR are listed in Table S5, S6, and S7, respectively.

### RNase R treatment

For RNase R treatment, 2 μg of total RNA was treated with 3 U/μg RNase R (Epicenter, USA) at 37 °C for 30 min. The treated RNA was reverse transcribed into cDNA and characterized by qPCR and agarose gel electrophoresis.

### Subcellular fractionation

Nuclear and cytoplasmic fractions were separated using a Nuclear and Cytoplasmic Protein Kit (Transgen Biotech, China). The subcellular localization of circATP9B and ATP9B mRNA was analyzed by qRT-PCR. GAPDH and U1 served as fractionation indicators.

### RNA pulldown and mass spectrometry

For RNA pulldown assays, Streptavidin Magnetic Beads (Invitrogen, USA) were washed and then incubated with 1 μM 3ʹ biotin-labeled circRNA probe (RiboBio, China) at 37 °C for 2 h. Then, the biotinylated beads were incubated with cell lysate at 4 °C overnight. The next day, the beads were washed three times with wash buffer (20 mM Tris-HCl,10 mM NaCl, 0.1% Tween-20), and enriched RNAs and proteins were collected from the beads. RNAs were collected using *TransZol* Up reagent (Transgen Biotech, China) while proteins were collected by boiling in loading buffer (Solarbio, China). Proteins were analyzed by SDS-PAGE and Coomassie brilliant staining and quantified by LC-MS/MS (PTM Biolabs, China). The sequences for RNA pulldown are listed in Table S8.

### RNA immunoprecipitation (RIP)

The RIP assay was performed as previously described [30]. Briefly, 50 μL of protein A/G magnetic beads (MCE, USA) was incubated with 5 mg of antibody in 100 μL of NT buffer (250 mM Tris-HCl, 750 mM NaCl, 5 mM MgCl_2_, 0.25% NP-40) at room temperature for 4 h. The antibody-coated beads were washed twice with NT buffer at 4 °C. Cell lysates were transferred to tubes containing antibody-coated beads and incubated at 4 °C overnight. The following day, the antibody-coated beads were washed three times with the NT buffer. RNA was then isolated and collected from the beads using *TransZol* Up reagent (Transgen Biotech, China) according to the manufacturer’s instructions.

### RNA fluorescence in situ hybridization

RNA fluorescence in situ hybridization was performed with a FISH kit (GenePharma, China) according to the manufacturer’s instructions. Briefly, cells were fixed at 40%–50% confluency in 4% paraformaldehyde and then permeabilized with 0.5% Triton X-100. After pre-hybridization at 37 °C for 30 min, the cells were hybridized with a Cy3-labeled circATP9B probe (Gene Pharma, China) at 37 °C overnight. The next day, the treated cells were washed three times with 2× SSC buffer at 60 °C, and three times with 2× SSC buffer at 37 °C. Nuclei were stained with DAPI. The sequences of the probes used for FISH are listed in Table S9.

### Immunofluorescence

Hela and HEK293T cells were cultured on coverslips to 50% confluency and treated or not with 4 Gy of irradiation. For γH2AX (Millipore, cat#05-636), Flag (Proteintech, cat#20543-1-AP), MYH9 (Proteintech, cat#60233-1-Ig), and HP1α (CST, cat#2616) antibody, cells were fixed in 4% paraformaldehyde for 20 min and permeabilized with 0.1% Triton X-100 at room temperature for 20 min. For the RAD51 (GeneTex, cat#100469) antibody, immunofluorescence staining was performed as previously described [31]. Briefly, cells were permeabilized with 0.5% Triton X-100 on ice for 5 min, fixed in 4% paraformaldehyde for 20 min, and incubated with the primary antibody at 4°C overnight. The next day, after washing three times with PBS, the cells were incubated with the secondary antibody at 37 °C for 1 h.

### Immunoprecipitation

For the Immunoprecipitation assay, cells were lysed in lysis buffer (20 mM Tris-KCl,150 mM NaCl,1% NP-40,1% sodium deoxycholate) for 30 min on ice, centrifuged at 16,000 × *g* for 10 min at 4 °C, and the collected lysate was incubated with antibody-coated beads (Sigma, Germany) at 4 °C overnight. The next day, the antibody-coated beads were washed three times with lysis buffer, boiled in loading buffer at 100 °C for 10 min, and subjected to western blot analysis.

### Western blot

Cells were treated with ice-cold RIPA buffer (50 mM Tris-KCl,150 mM NaCl,1% NP-40, 0.1% SDS and 1× cocktail protease inhibitor) for 10 min and cell lysates were collected by centrifugation at 13,000 × *g* for 10 min at 4 °C and boiled in loading buffer (Solarbio, China) for 10 min at 100 °C. Proteins were separated by SDS-PAGE and transferred to a polyvinylidene fluoride (PVDF) membrane (Millipore, USA) for further analyses with specific antibodies.

### CircRNA and protein interaction modeling

Prediction of RNA–protein interaction of circATP9B withMYH9 using the catRAPID algorithm [32]. The secondary structure of circATP9B was predicted using the RNAfold web server (http://rna.tbi.univie.ac.at/cgi-bin/RNAWebSuite/RNAfold.cgi), and RNAComposer (http://rnacomposer.cs.put.poznan.pl/) was used to generate its 3D structure. The structure of the MYH9 heavy chain was downloaded from the Protein Data Bank (PDB No.3zwh). Molecular docking between circATP9B and MYH9 was performed using the HDOCK web server and visualized by PyMol (version 3.0.3).

### Cycloheximide chase assay

Hela and HEK293T cells were cultured in 6-well plates for 24 h and then treated with 100 μg/mL cycloheximide, respectively. The cells were collected at the indicated times and prepared for western blot analysis.

### Cell viability assay

Cells transfected with the indicated siRNAs or plasmids were seeded in 96-well plates and treated with different concentrations of olaparib for 24 h. After removing the olaparib, 90 μL of fresh complete medium and 10 μL of CCK-8 reagent (Bioss, China) were added to each well. The plates were then incubated at 37 °C with 5% CO_2_ for 2 h. Finally, the absorbance of the solution in each well was measured at 450 nm using a scan reader (ALLSHENG, China).

### Live cell imaging

Hela cells stably expressing mCherry-RAD52, YFP-53BP1, or nuclear actin-chromobody Tag-GFP constructs were cultured on 35 mm glass-bottom microwell dishes (NEST, China). Cells were transfected with the indicated siRNAs or plasmids for 24 h and exposed to 4 Gy of irradiation. Images were acquired on an All-in-One Fluorescence microscopic Imaging System (Keyence, Japan), with a 60×/1.49 oil-immersion objective lens, an automated XY stage, and a chamber with 37 °C, 5% CO_2_ atmosphere. For imaging, 561 nm excitation was used for cells expressing mCherry-RAD52, while 488 nm excitation was employed for cells expressing YFP-53BP1 and GFP-nuclear actin.

### Imaging analysis in time-lapse experiments

DSB site movements were analyzed as previously described [33]. In brief, images of DSB sites were processed using Fiji/ImageJ (version 1.48) [19, 34]. At each time point, each z stack was projected onto a single *z* plane, and then the *t* stack was registered using the StackReg plug-in in ImageJ to correct for cell movement [35]. Single foci were tracked using the TrackMate plug-in in ImageJ [36]. The Mean Square Displacement (MSD) value was calculated using the formula ${MSD}=\, < {\left(x\left(t+\varDelta t\right)-x\left(t\right)\right)}^{2} >$, where $x$ is the DSB site location and $t$ is the time. The @msdanalyzer MATLAB (version 2024a) class was employed to analyze and visualize the resulting data [37]. The error bar for each point represents the weighted SEM over all MSD curves. The distance moved by foci was calculated as the cumulative distance relative to their starting position over 100 min, using the formula: $D\left(i\right)=\sqrt{{\left(X\left(i\right)-X\left(i-1\right)\right)}^{2}+{\left(Y\left(i\right)-Y\left(i-1\right)\right)}^{2}}$, where ${X}$ and ${Y}$ are the ${x}$ and ${y}$ coordinates of foci at time $i$. Movement velocity was derived from individual DSB site trajectories using the TrackMate plug-in. For analysis of DSB site clustering, a clustering event was defined as the colocalization of ≥ 2 foci over 10 min. Foci sizes (single-foci intensity) and nuclear actin filament intensities were quantified using ImageJ software.

### Micronucleus assay

Micronuclei were detected according to a previously described method [16]. Briefly, cells transfected with siRNAs or plasmids for approximately 24 h were exposed to 4 Gy of radiation, and, after 24 h, were stained with HP1α and DAPI for immunofluorescence analysis. Micronuclei were identified as DAPI-positive signals colocalizing with HP1α signals (heterochromatin marker) outside the main nuclear periphery.

### Statistics analysis

All experiments were repeated at least three times. Statistical analyses were performed using GraphPad Prism software (version 8.0.1) and significance is indicated in the figure legends. Data are expressed as means ± SEM or SD as indicated in the figure legends and were analyzed by the unpaired two-tailed Student’s *t*-test and one-way or two-way ANOVA with tests for multiple comparisons. *p* < 0.05 was considered statistically significant.

## Results

### SF3B1 mutation promotes circATP9B production

**Fig. 1:**
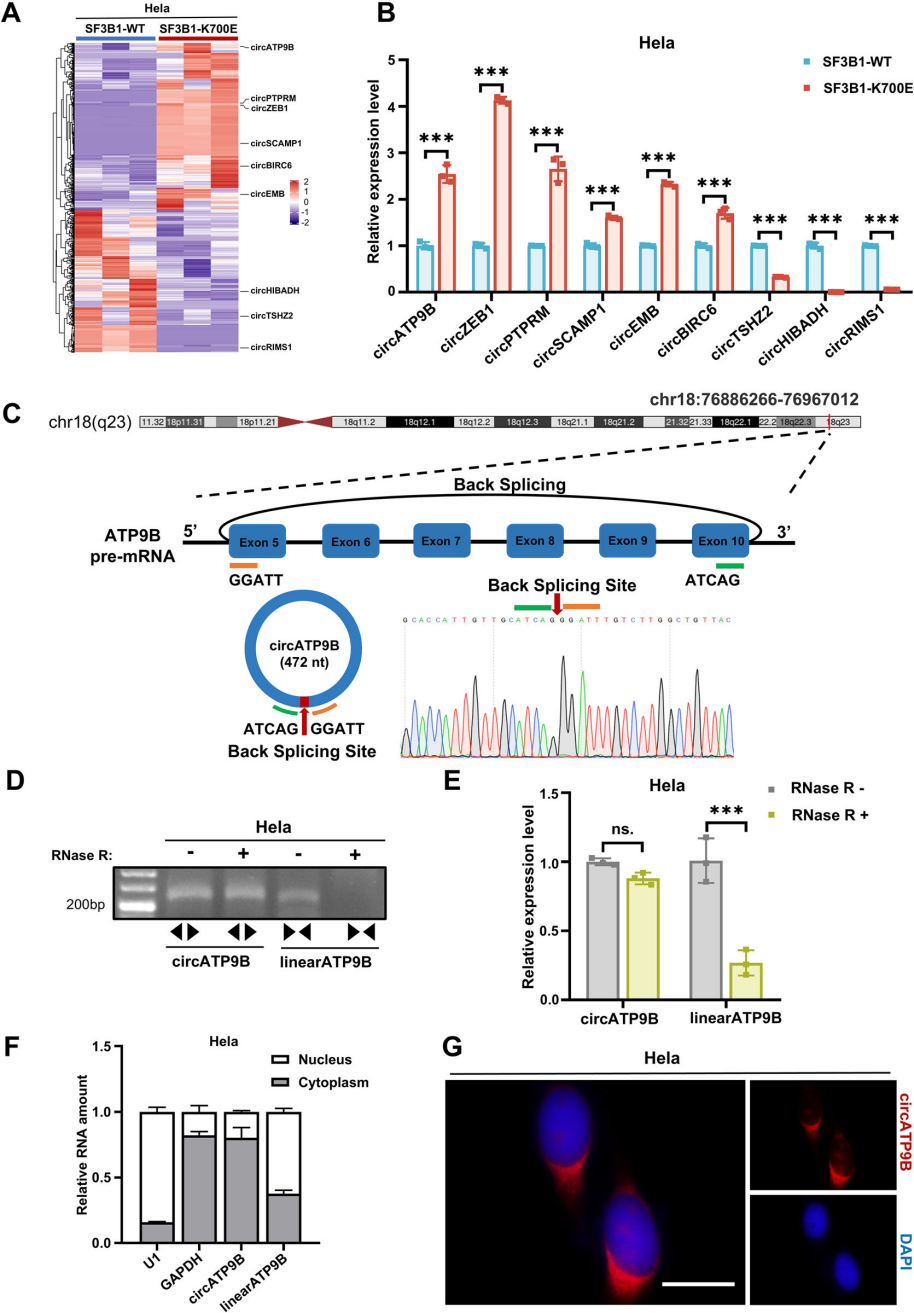
Identification and Characterization of circATP9B. **A** Heatmap of the differentially expressed circRNAs in Hela cells with or without SF3B1-K700E mutation (*n* = 3 independent samples). **B** qRT-PCR validation of the expression of some circRNAs in Hela cells with or without SF3B1-K700E mutation. **C** Schematic diagram of circATP9B. The back-splicing junction of circATP9B was identified by Sanger sequencing. **D** Agarose gel electrophoresis of the PCR products amplified from total RNA of Hela cells that were treated or non-treated with RNase R, using divergent primers for circATP9B and convergent primers for linearATP9B. **E** qRT-PCR analysis of the relative expression of circATP9B and linearATP9B in total RNA of Hela cells that were treated or non-treated with RNase R. **F** Nuclear and cytoplasmic fractions of Hela cells were separated, and relative amount of circATP9B and linearATP9B in nuclear and cytoplasmic RNA was analyzed by qRT-PCR. U1 and GAPDH were served as nuclear and cytoplasmic markers, respectively. **G** RNA fluorescence in situ hybridization assay to show the subcellular localization of circATP9B (red) in Hela cells. Nuclei were stained with DAPI (blue), scale bar = 10 μm. Data are expressed as the mean ± SD, one-way ANOVA with multiple comparisons (**B**,**E**,**F**). ns non-significant, * *p* < 0.05, ** *p* < 0.01, *** *p* < 0.001.

SF3B1-K700E heterozygous mutation and isogeneic wild-type (WT) Hela and K562 cells were generated using CRISPR-Cas9 technology (Fig. S1). To investigate whether SF3B1-K700E mutation affects circRNA biogenesis, we undertook a circRNA-seq analysis on Hela cells and identified 1416 differentially expressed circRNAs ( | fold change | ≥ 2, *p* < 0.05) between cells carrying SF3B1-K700E mutation and WT cells, of which 766 were upregulated and 650 were downregulated in the former (Fig. 1A). Some of these differentially expressed circRNAs were validated by qRT-PCR (Fig. 1B). We also conducted a circRNA-seq analysis in HEK293T cells transfected with constructs expressing wildtype or K700E-mutated SF3B1 and identified 1256 differentially expressed circRNAs, 713 of which showed increased expression while 543 displayed decreased expression in cells expressing SF3B1-K700E (Supplementary Fig. 2A). In total, 29 circRNAs were found to be dysregulated in both Hela and HEK293T cells harboring SF3B1-K700E mutation, with circATP9B showing the most robust increase in expression levels (Fig. 1B, Supplementary Fig 2B). Increased expression of circATP9B was also detected in K562 cells with SF3B1-K700E mutation (Supplementary Fig. 2C). Moreover, the SF3B1 mutation did not alter the expression of ATP9B pre-mRNA and mRNA, suggesting that the increased expression of circATP9B in cells with SF3B1 mutation is caused by altered RNA splicing, but not expression of ATP9B pre-mRNA (Supplementary Fig. 2D, E). Furthermore, we analyzed previously published sequencing data from patients with MDS and found that expression of circATP9B was significantly increased in patients with SF3B1 mutation compared with patients without SF3B1 mutation (Supplementary Fig. 2F) [38]. CircATP9B was derived from back-splicing of exon 5 to exon 10 of ATP9B pre-mRNA (chr18:76886266-76967012) (Fig. 1C) and remained stable after RNase R treatment (Fig. 1D, E, Supplementary Fig. 2G, H). Cellular fractionation and fluorescence in situ hybridization assays demonstrated that circATP9B was predominantly localized to cytoplasm (Fig. 1F, G, Supplementary Fig. 2I, J). Taken together, these results demonstrated that SF3B1-K700E mutation influences the biogenesis of circRNAs, including circATP9B.

### CircATP9B promotes the degradation of MYH9

**Fig. 2:**
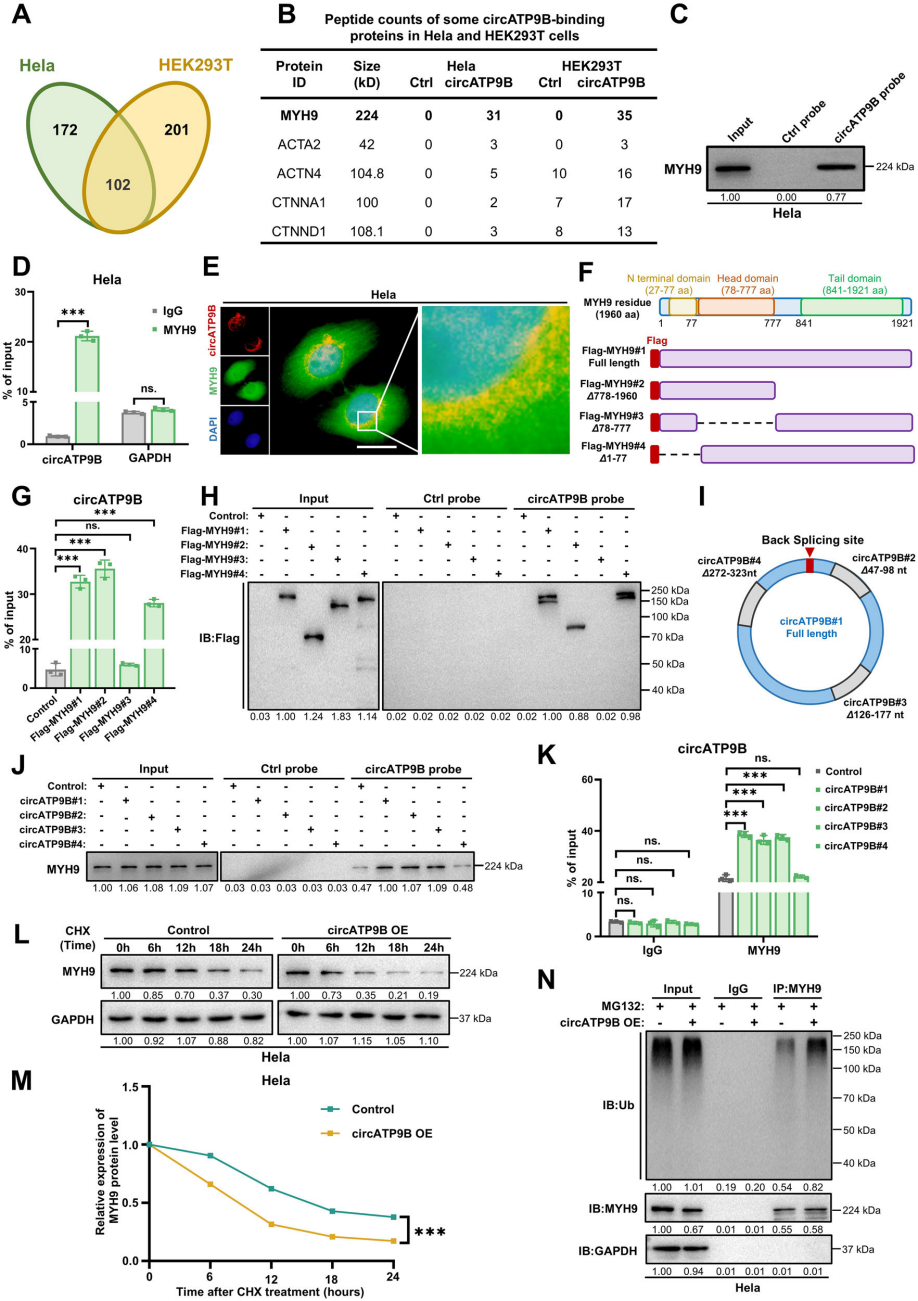
CircATP9B promotes MYH9 degradation *via* ubiquitin-proteasome pathway. **A** A Venn diagram showing the numbers of circATP9B-binding proteins in Hela and HEK293T cells that were pulled down by circATP9B probe and identified by LC-MS/MS. **B** Peptide counts from LC-MS/MS analysis of some circATP9B-binding proteins that were identified in both Hela and HEK293T cells. **C** RNA pulldown using circATP9B and control probes (Ctrl probe) and western blot analysis to confirm the interaction between circATP9B and MYH9 in Hela cells. **D** RNA immunoprecipitation analysis using MYH9 antibody and IgG as control to confirm the interaction between circATP9B and MYH9 in Hela cells. GAPDH was used as control. **E** Representative immunofluorescence images of the localization of circATP9B (red) and MYH9 (green) in Hela cells. Nuclei were stained with DAPI (blue), scale bar = 10 μm. **F** Schematic diagram of full-length and truncated MYH9 protein. **G** RIP assays with anti-Flag antibody in HEK293T cells transfected with MYH9 constructs. Co-precipitated RNAs were analyzed by qRT-PCR. **H** RNA pulldown assays using biotin-labeled circATP9B probe and Ctrl probe in HEK293T cells expressing full-length or truncated MYH9, co-precipitated proteins were detected by immunoblots using anti-Flag antibodies. **I** Schematic illustration of the potential binding sequences of MYH9 in circATP9B and circATP9B expression constructs with indicated deletion were shown. **J** RNA pulldown assays using biotin-labeled Ctrl probe and circATP9B probe in HEK293T cells transfected with indicated circATP9B expression constructs. Co-precipitated proteins were detected by immunoblots using anti-MYH9 antibodies. **K** RIP assays with anti-MYH9 antibody in HEK293T cells transfected with indicated circATP9B expression constructs. Co-precipitated RNAs were analyzed by qRT-PCR. **L** Western blots to show the expression of MYH9 in Hela cells with or without circATP9B overexpression and treated with 100 μM cycloheximide at the indicated times. GAPDH was used as control. **M** Quantification of the relative expression of MYH9 protein through calculating the intensities of western blot bands shown in (**L**). **N** Western blot to show ubiquitination level of MYH9 in circATP9B overexpression or control Hela cells treated with 20 μM MG132 for 12 h. Data are expressed as the mean ± SD (**D**,**G**,**K**,**M**), one-way ANOVA (**D**,**G**,**K**) and two-way ANOVA with multiple comparisons (**M**). ns, non-significant, *** *p* < 0.001.

To explore the function of circATP9B, we performed a pulldown assay with a probe targeting its back-splicing junction (Supplementary Fig. 3A–D). Through LC-MS/MS analysis, we identified 274 proteins in Hela cells and 303 proteins in HEK293T cells that specifically interacted with the circATP9B probe (Fig. 2A). Of these, 102 were shared by both cell lines, with MYH9 showing the highest peptide count (Fig. 2B and Supplementary Fig. 3E). Western blot analysis confirmed that MYH9 was specifically pulled down with the circATP9B probe (Fig. 2C and Supplementary Fig. 3F). Reciprocally, MYH9 antibody also pulled down circATP9B (Fig. 2D and Supplementary Fig. 3G). Furthermore, circATP9B and MYH9 were colocalized in the cytoplasmic region of Hela and HEK293T cells mostly (Fig. 2E and Supplementary Fig. 3H). Together, these results demonstrated that circATP9B interacts with MYH9.

To identify the specific domain in MYH9 that mediate the interaction between MYH9 and circATP9B, we created truncated forms of MYH9, including MYH9 ΔTail domain (Δ778–1960), MYH9 ΔHead domain (Δ78–777), and MYH9 ΔN terminal domain (Δ27–77), and found that the head domain was required for circATP9B binding (Fig. 2F–H, Supplementary Fig. 3I–L). Moreover, HDOCK was used to predict the binding sequence for MYH9 in circATP9B (Supplementary Fig. 3M), and deletion of the predicted binding sequence (circATP9B Δ272–323 nt), but not other random sequences (circATP9B Δ47–98 nt and circATP9B Δ126–177 nt), impaired the interaction between circATP9B and MYH9 (Fig. 2I–K, Supplementary Fig. 3N–Q).

To investigate the potential functional consequences of the interaction between circATP9B with MYH9, we synthesized a circATP9B expression plasmid and siRNAs targeting circATP9B (Supplementary Fig. 3R-3S and 4A-4B). We noted that while overexpression or knockdown circATP9B did not affect expression of MYH9 mRNA (Supplementary Fig. 3T-3U and 4C), it affected expression of MYH9 protein (Supplementary Fig. 3V-3W and 4D). Furthermore, results from cycloheximide treatment showed that the stability of MYH9 protein was decreased in cells with circATP9B overexpression (Fig. 2L-M and Supplementary Fig. 3X-Y), whereas the stability of MYH9 protein was increased in cells with circATP9B knockdown (Supplementary Fig. 4E-4H). In addition, circATP9B overexpression promoted the ubiquitination of MYH9, suggesting that circATP9B overexpression enhances MYH9 degradation through increased ubiquitination (Fig. 2N, Supplementary Fig. 3Z, 4I, J).

### MYH9 is essential for the organization of nuclear actin filaments during DNA repair

**Fig. 3:**
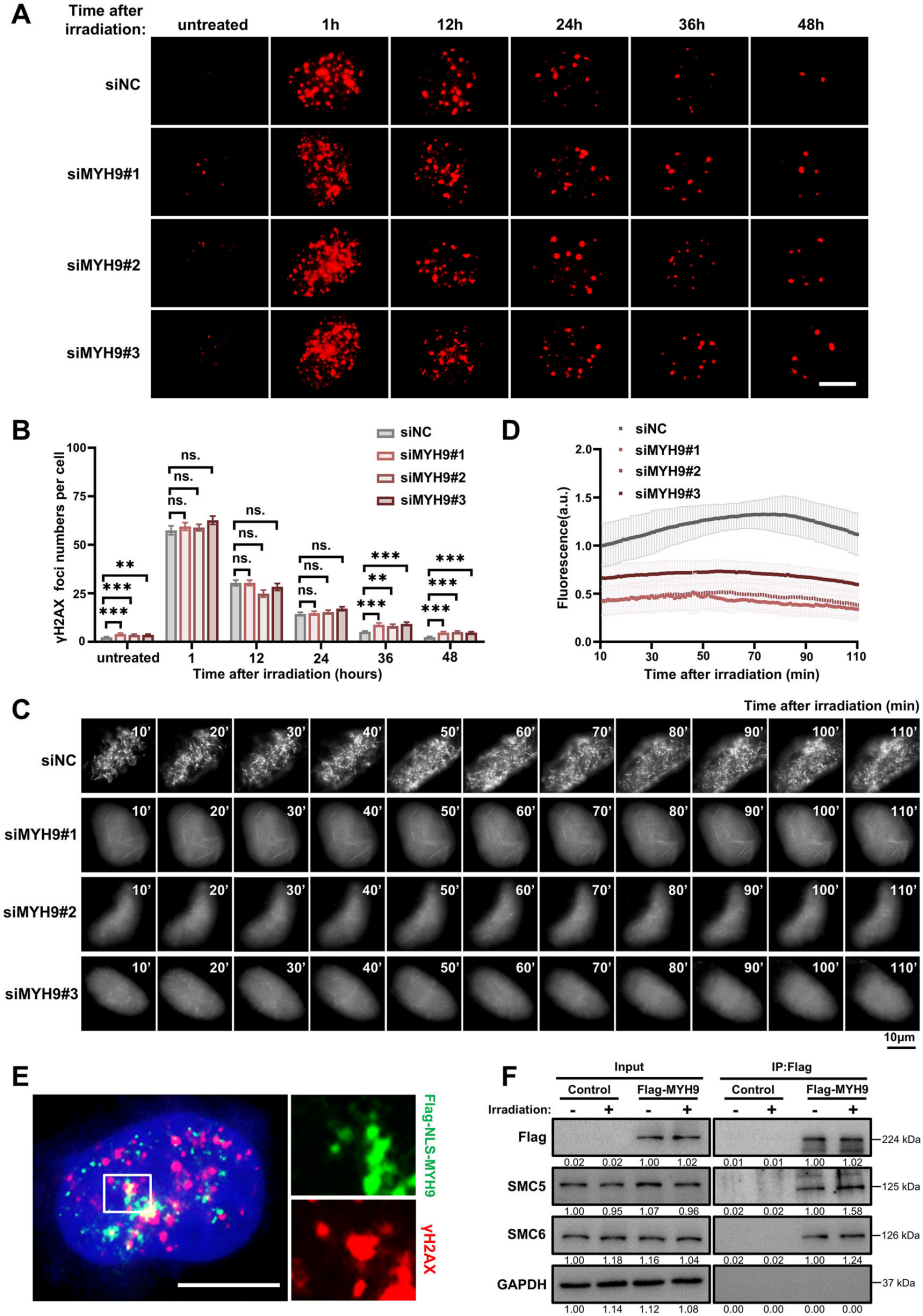
MYH9 is required for the organization of nuclear actin filaments during DNA repair. **A** Representative immunofluorescence images of γH2AX foci in Hela cells transfected with control siRNA (siNC) or MYH9 siRNA (siMYH9) at indicated times after irradiation were shown. γH2AX was stained with red. Scale bar = 10 μm. **B** Quantification of γH2AX foci at different times after irradiation (50 cells per group). **C** Representative images of nuclear actin filaments in siNC and siMYH9 Hela cells and treated with irradiation. Scale bar = 10 μm. **D** Quantification of the intensities of nuclear actin filaments in siNC and siMYH9 Hela cells images as shown in (**C**) (*n* ≥ 15). **E** Representative images of immunofluorescence to show the localization of γH2AX (red) and Flag-NLS-MYH9 (green) after irradiation. Nuclei were stained with DAPI (blue), scale bar = 10 μm. **F** Western blot analysis of proteins immunoprecipitated with anti-Flag antibody in Hela cells transfected with Flag-MYH9 or control vectors and treated or non-treated with irradiation. Data are expressed as the mean ± SEM (**B**) or SD (**D**), unpaired two-tailed Student’s *t*-test (**B**) and two-way ANOVA with multiple comparisons (**D**). ns non-significant, * *p* < 0.05, ** *p* < 0.01, *** *p* < 0.001.

MYH9 knockdown has been reported to lead to increased DNA damage in prostate cancer cells and squamous cell carcinomas [28, 29], and we confirmed that MYH9 knockdown also led to an increase in the number of γH2AX foci in Hela and HEK293T cells, indicative of impaired DNA repair (Supplementary Fig. 5). Furthermore, while MYH9 knockdown Hela cells exhibited similar numbers of γH2AX foci as control cells at 1, 12, and 24 h post-irradiation, the number of γH2AX foci was elevated 36 and 48 h after irradiation in MYH9-deficient Hela cells (Fig. 3A, B), demonstrating that such impaired DNA repair leads to the accumulation of γH2AX foci at later stages upon DNA damage infliction. Similarly, MYH9 knockdown Hela cells showed increased numbers of RAD51 foci starting at 24 h after irradiation (Supplementary Fig. 6A, B), suggesting that HDR was impaired in these cells. Moreover, MYH9 knockdown enhanced the sensitivity of cells to olaparib, a PARP inhibitor that suppresses DNA single-strand breaks (SSBs) repair [39], indicating that there was a synthetic lethal effect from MYH9 knockdown and olaparib treatment (Supplementary Fig. 6C).

As MYH9 is essential for organization of actin filaments in cytoplasm [25–27], we investigated whether it plays a similar role in nucleus during DNA repair. A GFP-tagged and NLS (nuclear localization signaling) -modified F-actin chromobody was used to visualize the dynamic polymerization of nuclear actin filaments in irradiation-treated Hela cells (Supplementary Fig. 6D–F). Strikingly, we found that MYH9 knockdown significantly reduced the irradiation-induced organization of nuclear actin network (Fig. 3C, D, Supplementary Video 1A-1D). In addition, MYH9 was colocalized with γH2AX (Fig. 3E), and the binding of MYH9 to SMC5, a factor with an essential role in homologous recombination [40], was enhanced after irradiation, suggesting that MYH9 might be recruited to DNA repair sites through SMC5 (Fig. 3F and Supplementary Fig. 6G). Combined, these results demonstrated that MYH9 play an important role in the organization of nuclear actin filaments during DNA repair.

### MYH9 promotes the mobility and clustering of DSBs undergoing HDR

**Fig. 4:**
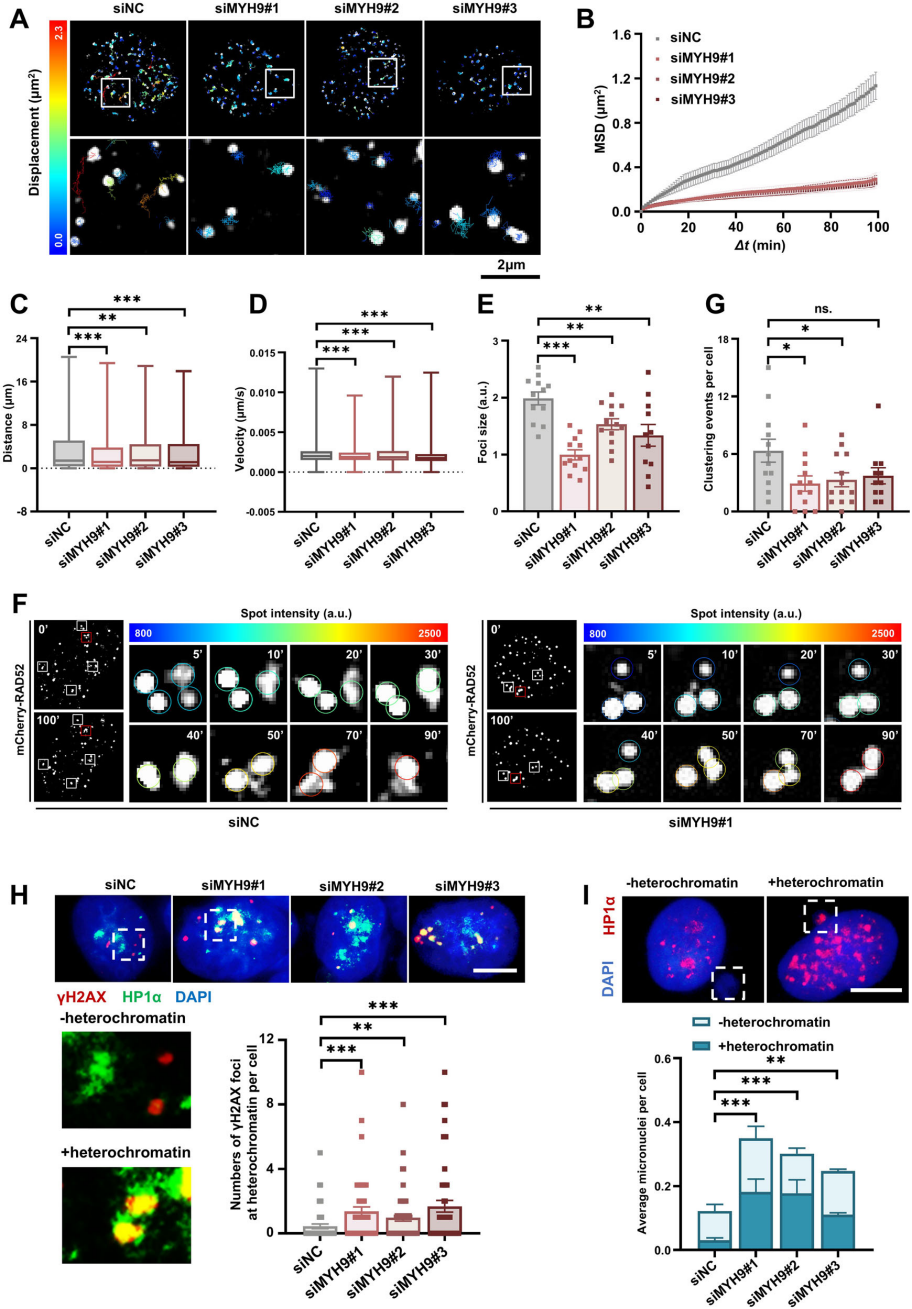
MYH9 regulates the movement and clustering of DSB sites. **A** Representative images of traces of mCherry-RAD52 foci in siNC and siMYH9 Hela cells over 100 min after irradiation. Enlarged images were shown below (boxed). Scale bar = 2 μm. **B–E** Mean-square displacement (MSD) (**B**), distance traveled (**C**), velocity (**D**, sizes (**E**) of mCherry-RAD52 foci in siNC and siMYH9 Hela cells treated with irradiation. 2415 foci from 12 nuclei of siNC,1275 foci from 12 nuclei of siMYH9#1, 1818 foci from 13 nuclei of siMYH9#2, 1774 foci from 11 nuclei of siMYH9#3. *Δt*, time intervals. **F** Representative images of mCherry-RAD52, with boxes indicating clustering of mCherry-RAD52 foci in siNC (left) and siMYH9#1 (right) Hela cell. Enlarged views of clustering events over time were presented from left to right (red box). Circles represent foci intensity. **G** mCherry-RAD52 foci clustering events in siNC and siMYH9 Hela cells treated with irradiation. **H** Immunofluorescence and quantification of γH2AX foci associated with HP1α in Hela cells transfected with siNC or siMYH9 at 48 h after irradiation, and 50 cells per group were used for quantification. γH2AX was stained with red, HP1α was stained with green, nuclei were stained with DAPI (blue), scale bar = 10 μm. I Immunofluorescence and quantification of micronuclei stained with HP1α in siNC an siMYH9 Hela cells at 48 h after irradiation (*n* > 140 cells). Data are expressed as the mean ± SEM (**B–E**,**G–I**), two-way ANOVA (**B**) and one-way ANOVA with multiple comparisons (**C–E**,**G–I**). ns, non-significant, * *p* < 0.05, ** *p* < 0.01, *** *p* < 0.001.

As clustering of DSBs enhances efficiency of HDR [18, 19], we tested the role of MYH9 in the movement and clustering of DSBs in Hela cells. DSBs undergoing HDR were visualized using mCherry-tagged RAD52 and those repaired *via* NHEJ were visualized using YFP-tagged 53BP1 (Supplementary Fig. 7A). The movements of these DSBs were tracked and the biophysical properties of these movements were analyzed by calculating mean-square displacement (MSD). The MSD, distance moved, and velocity of RAD52 foci were significantly reduced following MYH9 knockdown (Fig. 4A–D and Supplementary Video 1E-1H). Furthermore, MYH9 knockdown significantly decreased the frequency of clustering events and blocked the increase in the size of RAD52 foci (Fig. 4E–G). In contrast, the MSD, distance moved, and velocity of 53BP1 foci were not affected by MYH9 knockdown, suggesting that NHEJ-mediated DNA repair was not impacted by MYH9 deficiency (Supplementary Fig. 7B–E and supplementary Video 1I-1L). This was consistent with previous studies showing that nuclear actin filaments are not required for the movement of DSB sites undergoing NHEJ-mediated repair [19]. Notably, MYH9 knockdown led to an increase in the number of γH2AX foci in HP1α-labeled heterochromatin domains and resulted in the formation of heterochromatic micronuclei after irradiation (Fig. 4H, I), which demonstrated that MYH9 deficiency leads to the accumulation of detrimental effects on heterochromatin.

Caridi et al. [18]. reported that MYO1A and MYO1B are essential for the trafficking of DSBs undergoing HDR along nuclear actin filaments. Therefore, we compared the effects of MYO1A, MYO1B, and MYH9 knockdown on the mobility of DNA repair foci and organization of nuclear actin in Hela cells. MYO1A, MYO1B, or MYH9 knockdown increased the number of γH2AX and RAD51 foci after irradiation and enhanced cell sensitivity to olaparib treatment (Supplementary Fig. 8). As with MYH9 knockdown, MYO1A and MYO1B knockdown did not impact the mobility of 53BP1 foci (Supplementary Fig. 9 and Supplementary Video 2A–D). As reported by Caridi et al. [18], MYO1A and MYO1B knockdown suppressed the movement of RAD52 foci, led to the retention of γH2AX foci in heterochromatin, and increased the number of heterochromatin-associated micronuclei after irradiation; however, their effects were less pronounced than those observed with MYH9 knockdown (Supplementary Fig. 10A–H and supplementary Video 2E–H). Strikingly, in contrast to MYH9 knockdown, MYO1A or MYO1B deficit did not lead to reduced organization of nuclear actin network (Supplementary Fig. 10I, J, Supplementary Video 2I–L). Together, these results demonstrated that MYH9, but not MYO1A or MYO1B, enhanced the mobility and clustering of DSBs undergoing HDR by organizing nuclear actin filaments.

### CircATP9B overexpression decreases the efficiency of DNA repair through MYH9

**Fig. 5:**
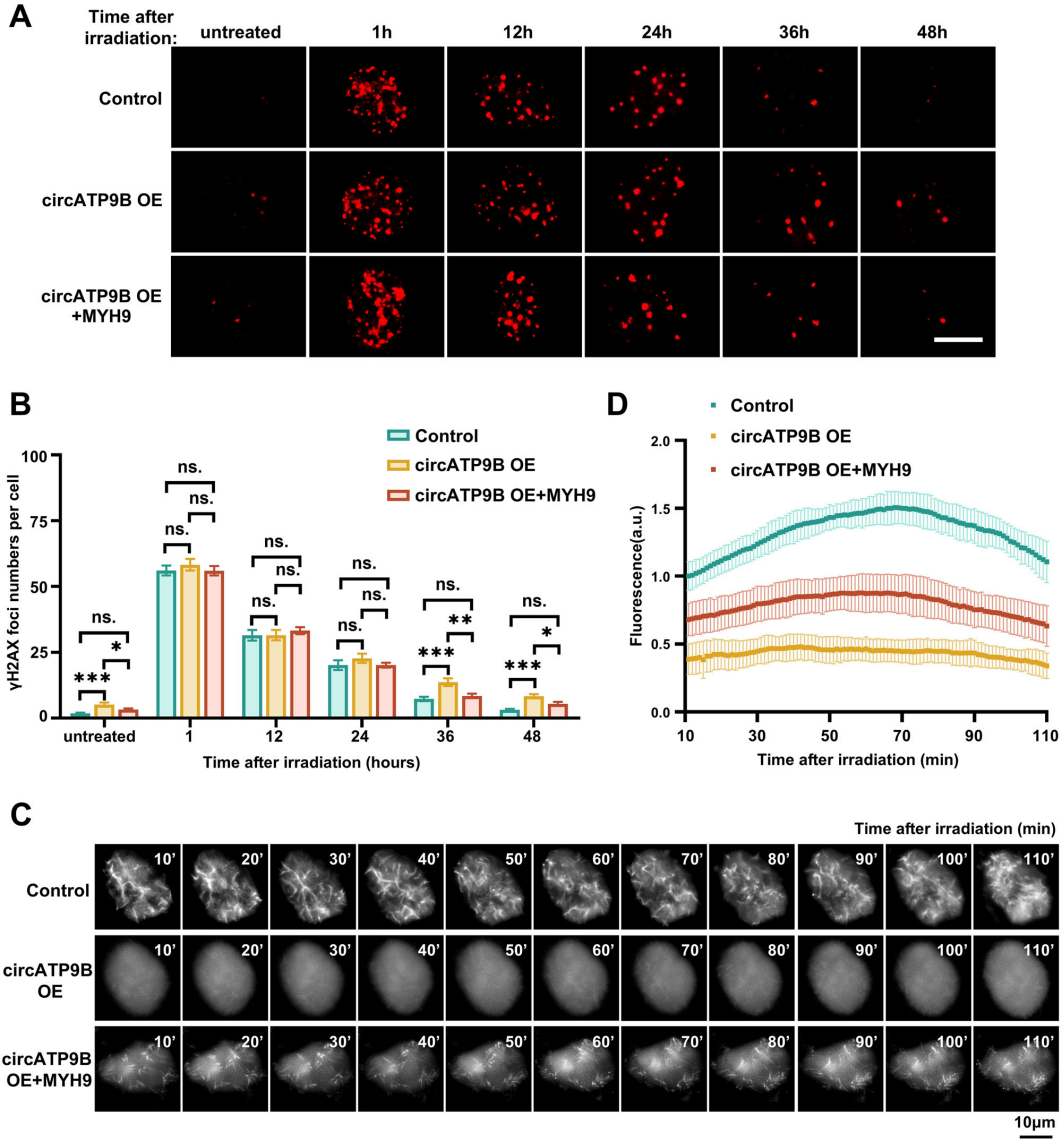
MYH9 overexpression rescues DNA repair defects caused by circATP9B. **A** Representative immunofluorescence images of γH2AX in Hela cells with control, circATP9B overexpression (circATP9B OE), circATP9B and MYH9 overexpression (circATP9B OE + MYH9) at indicated times after 4 Gy irradiation. γH2AX was stained with red, scale bar = 10 μm. **B** Quantification of γH2AX foci as shown in (**A**) after irradiation (50 cells per group). **C** Representative images of nuclear actin filaments in control, circATP9B OE, circATP9B OE + MYH9 Hela cells at indicated times after irradiation. **D** Quantification of fluorescence intensities from nuclear actin filaments (*n* ≥ 15). Data are expressed as the mean ± SEM (**B**) or SD (**D**), unpaired two-tailed Student’s *t*-test (**B**) and two-way ANOVA with multiple comparisons (**D**). ns, non-significant, * *p* < 0.05, ** *p* < 0.01, *** *p* < 0.001.

The fact that circATP9B interacts with MYH9 suggested that circATP9B might affect DNA repair through MYH9. Thus, we investigated whether circATP9B influences DNA repair by examining the effects of its overexpression on the formation and clustering of DSBs organization of nuclear actin filaments, nuclear actin organization, and sensitivity to olaparib treatment. We found that circATP9B overexpression resulted in an increase in the number of γH2AX foci (Supplementary Fig. 11A–C). Importantly, overexpression of MYH9 led to a decline in the number of γH2AX foci in Hela cells with circATP9B overexpression (Fig. 5A, B and Supplementary Fig. 11D–F). Similarly, circATP9B overexpression led to the retention of RAD51 foci at 24 h and beyond after irradiation, while MYH9 overexpression suppressed the increase in the number of RAD51 foci in Hela cells with circATP9B overexpression (Supplementary Fig. 11G, H). Moreover, MYH9 overexpression alleviated the sensitivity of Hela cells with circATP9B overexpression cells to olaparib treatment (Supplementary Fig. 11I) and restored the organization of nuclear actin filaments in Hela cells with circATP9B overexpression (Fig. 5C, D and supplementary Video 3A–C).

**Fig. 6:**
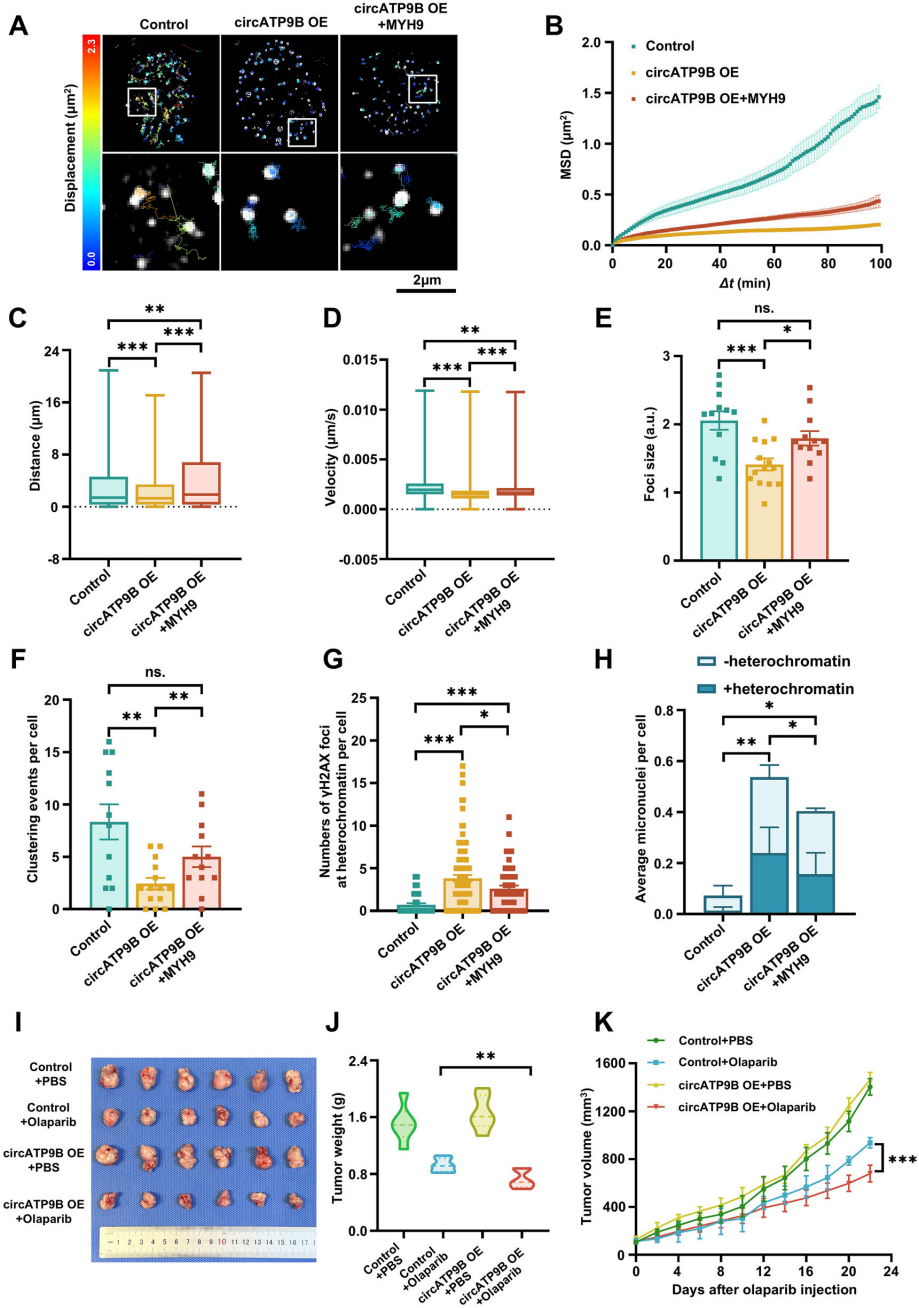
CircATP9B overexpression suppresses relocalization and repair of DSB sites. **A** Representative images of mCherry-RAD52 foci traces in control, circATP9B OE, circATP9B OE + MYH9 cells over 100 min after 4 Gy irradiation. Scale bar = 2 μm. B–F MSD (**B**), distance (**C**), velocity (**D**), sizes (**E**), and clustering events (**F**) of mCherry-RAD52 foci in control, circATP9B OE, circATP9B OE + MYH9 cells. 2207 foci from 12 nuclei of control,1621 foci from 14 nuclei of circATP9B OE, 1747 foci from 12 nuclei of circATP9B OE + MYH9. *Δt*, time intervals. **G**,**H** Numbers of γH2AX foci (**G**) and micronuclei associated with HP1α (**H**) in control, circATP9B OE, circATP9B OE + MYH9 cells at 48 h after 4 Gy irradiation (50 cells per group for γH2AX foci, *n* > 110 cells per group for micronuclei). **I** Images of tumors obtained from BALB/c nude mice implanted with control or circATP9B OE cells and treated or non-treated with olaparib. **J**,**K** Quantification of weight (**J**) and volume (**K**) of tumors as shown in (**I**) (*n* = 6). Data are expressed as the mean ± SEM (**B–H**) or SD (**J**,**K**), one-way ANOVA (**C–H**,**J**) and two-way ANOVA with multiple comparisons (B,K). ns, non-significant, * *p* < 0.05, ** *p* < 0.01, *** *p* < 0.001.

Importantly, while circATP9B overexpression reduced the mobility, clustering, and sizes of RAD52 foci, MYH9 overexpression suppressed these effects in Hela cells with circATP9B overexpression (Fig. 6A–F and Supplementary Video 3D–F). In contrast, circATP9B overexpression did not influence the mobility of YFP-53BP1, suggesting that NHEJ-based repair was not affected (Supplementary Fig. 12A–D, supplementary Video 3G–I). Moreover, the numbers of γH2AX foci and micronuclei in heterochromatin increased significantly in Hela cells with circATP9B overexpression, implying that DNA repair and chromosome rearrangements in heterochromatic regions were impaired in these cells (Fig. 6G, H). To investigate the effects of circATP9B in vivo, we constructed a Hela cell line stably overexpressing circATP9B and established xenograft tumors through subcutaneous injection of these cells into nude mice (Supplementary Fig. 12E). Olaparib treatment significantly decreased the size and weight of tumors formed by circATP9B-overexpressing Hela cells, demonstrating that circATP9B overexpression enhanced the sensitivity of tumors to olaparib (Fig. 6I–K). Taken together, these results indicated that circATP9B inhibits DNA repair through MYH9.

### CircATP9B mediates the deficits in DNA repair caused by SF3B1 mutation

**Fig. 7:**
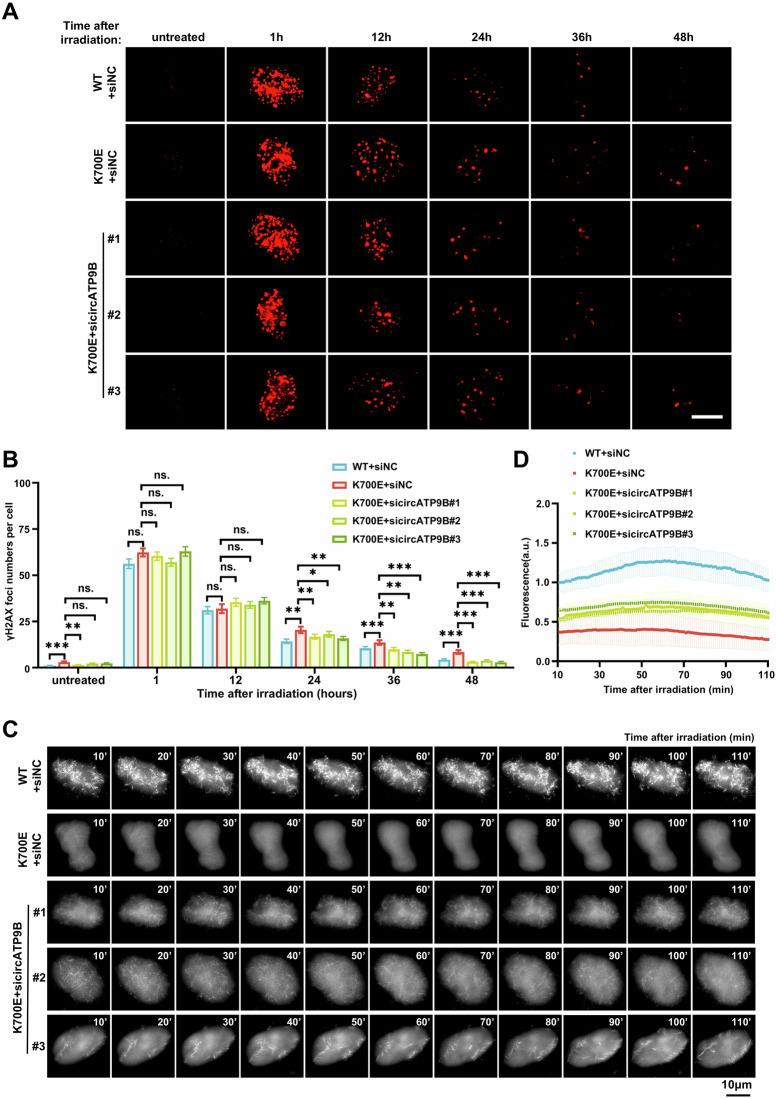

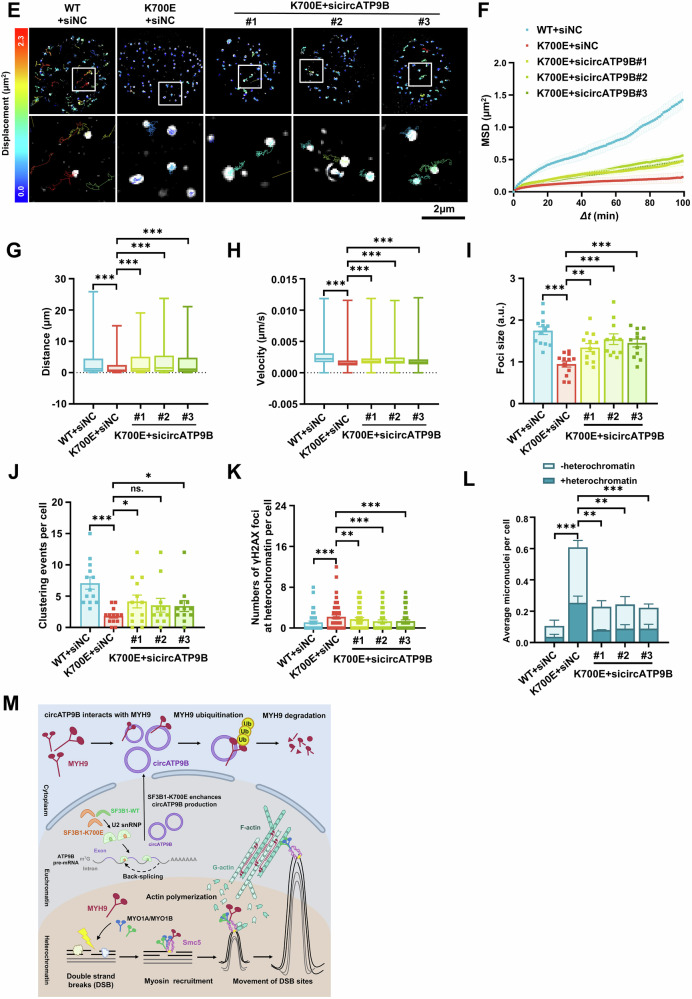
CircATP9B knockdown alleviates the deficits in DNA repair caused by SF3B1 mutation. **A** Representative immunofluorescence images of γH2AX foci in wildtype SF3B1 and control siRNA transfected (WT+siNC), SF3B1-K700E mutated and control siRNA transfected (K700E+siNC), SF3B1-K700E mutated and circATP9B siRNA transfected (K700E+sicircATP9B) Hela cells at indicated times after irradiation. γH2AX was stained with red, scale bar = 10 μm. **B** Quantification of γH2AX foci as shown in (**A**) (50 cells per group). **C** Representative images of the organization of nuclear actin filaments in WT+siNC, K700E+siNC, K700E+sicircATP9B Hela cells at indicated times after 4 Gy irradiation. **D** Quantification of fluorescence intensities from nuclear actin filaments (*n* ≥ 15). **E** Representative images of mCherry-RAD52 foci traces in WT+siNC, K700E+siNC, K700E+sicircATP9B Hela cells over 100 min after irradiation. Scale bar = 2 μm. **F–J** MSD (**F**), distance (**G**), velocity (**H**), sizes (**I**), and clustering events (**J**) of mCherry-RAD52 foci in WT+siNC, K700E+siNC, K700E+sicircATP9B Hela cells after irradiation. 1899 foci from 13 nuclei of WT+siNC, 1468 foci from 13 nuclei of K700E+siNC, 2034 foci from 13 nuclei of K700E+sicircATP9B#1, 1401 foci from 11 nuclei of K700E+sicircATP9B#2, 1689 foci from 12 nuclei of K700E+sicircATP9B#3. *Δt*, time intervals. **K–L** Numbers of γH2AX foci (**K**) and micronuclei associated with HP1α (**L**) in WT+siNC, K700E+siNC, K700E+sicircATP9B Hela cells at 48 h after irradiation (50 cells per group for γH2AX foci, *n* > 120 cells per group for micronuclei). **M** Schematic illustration of circATP9B molecular mechanism. Data shown as mean ± SEM (B and F-L) or SD (**D**), unpaired two-tailed Student’s *t*-test (**B**), one-way ANOVA (**G–L**) and two-way ANOVA with multiple comparisons (**D** and **F**). ns, non-significant, * *p* < 0.05, ** *p* < 0.01, *** *p* < 0.001.

Because SF3B1-K700E mutation upregulates the expression of circATP9B, we investigated whether circATP9B knockdown could reverse the deficits in DNA repair that occur in Hela cells harboring mutated SF3B1. After irradiation, circATP9B knockdown reduced the number of γH2AX foci in Hela cells with SF3B1-K700E mutation (Fig. 7A, B). Similarly, circATP9B knockdown reduced the number of RAD51 foci in Hela cells with SF3B1-K700E mutation at 24 h and later post-irradiation (Supplementary Fig. 13A, B). CircATP9B knockdown in Hela cells harboring SF3B1-K700E mutation also led to increased organization of nuclear actin network and enhanced cell survival following olaparib treatment (Fig. 7C, D, Supplementary Fig. 13C and supplementary Video 4A–E). Furthermore, while circATP9B knockdown restored the mobility and clustering of RAD52 foci in Hela cells with SF3B1-K700E mutation (Fig. 7E–J and Supplementary Video 4F–J), it did not affect the movement of 53BP1 foci (Supplementary Fig. 13D–G and supplementary Video 4K–O). Importantly, circATP9B knockdown in Hela cells with SF3B1-K700E mutation resulted in a significant reduction in the number of persistent γH2AX foci and micronuclei at heterochromatin domains (Fig. 7K, L). Overall, these results demonstrated that circATP9B mediates the DNA repair defect caused by SF3B1 mutation.

## Discussion

While it is well established that mutations in SF3B1 promote cancer progression through disruption of mRNA splicing, our study has shown that they also lead to dysregulated expression of circRNAs, which contributes to cancer progression. Specifically, we found that SF3B1 mutation leads to increased expression of circATP9B, and, consequently, enhanced MYH9 degradation. In turn, MYH9 deficiency diminishes the movement and clustering of DSBs undergoing HDR, resulting in impaired DNA repair (Fig. 7M).

CircRNAs are generated through back-splicing, a process catalyzed by canonical RNA splicing machinery [22, 23]. For instance, SF3B1 deficiency has been shown to promote circRNA expression massively in Drosophila [23]. In contrast to SF3B1 knockdown, our study revealed that SF3B1 mutation affected the expression of a relatively small number of circRNAs. This aligns with findings from a study comparing myelodysplastic neoplasm samples from patients with or without SF3B1 mutation [38], which showed that SF3B1 mutation does not affect global circRNA production. These observations suggest that SF3B1 mutation affects circRNA expression differently from SF3B1 deficiency. Notably, another study found that mutations in U2AF1, another RNA splicing factor that is frequently mutated in cancer, lead to an increase in global circRNA levels, implying that U2AF1 mutation and SF3B1 mutation might influence circRNA expression through different mechanisms [24]. Studies have demonstrated that mutated SF3B1 tends to use more conserved branch point sequences (BPSs) with low minimum free energy binding to U2 snRNA for the splicing of pre-mRNA [41, 42]. Interestingly, a recent study by Damianov et al. found that although SF3B1 mutations can extensively alter the usage of BPS, not all such changes lead to the occurrence of alternative splicing events [43]. We analyzed the potential BPSs in the flanking intron of circATP9B, and found that they show similar minimum free energy as BPSs in other introns of ATP9B pre-mRNA, suggesting that the increased circATP9B production in cells with SF3B1-K700E mutation might not cause by the preferences of conserved BPSs. Further investigations are required to reveal the mechanism through which SF3B1-K700E mutation affects circRNA expression.

CircRNAs are expressed in a tissue- and cell-type-specific manner [44–46]. Here, we found that circRNA expression markedly differed between Hela and HEK293T cells. Nevertheless, circATP9B was expressed in both cell lines as well as in K562 cells, and SF3B1 mutation led to an increase in circATP9B expression in all three cell lines, highlighting the importance of circATP9B in multiple cell types. In addition, we analyzed the expression of circATP9B in previously published sequencing data of patients with MDS and found that it was also increased in patients harboring SF3B1-K700E mutation, suggesting that circATP9B might be involved in the progression of MDS [38]. Furthermore, through pan-cancer analysis, we found that circATP9B is highly expressed in several hematological malignancies such as AML, MBL, and ALL, indicating that circATP9B may serve as a potential biomarker of hematological malignancies.

CircRNAs play key roles in cancer progression through diverse mechanisms, including by serving as microRNA sponges, protein decoys, scaffolds, and templates for translation [47, 48]. We found that circATP9B interacts with and promotes the ubiquitination and degradation of MYH9. Multiple studies have demonstrated that circRNAs promote cancer progression by influencing protein stability [49, 50]. In breast cancer cells, circFOXO3 has been shown to bind to TP53 and a E3 ubiquitin ligase MDM2, thereby facilitating the ubiquitination and degradation of the former [51]. Similarly, circNDUFB2 was reported to interact with IGF2BPs and enhance their degradation by recruiting ubiquitin ligase TRIM25 [52]. Similarly, in hepatocellular carcinoma, it was found that circFADS1 interacts with GSK3β and promotes its ubiquitination and degradation by recruiting ubiquitin ligase RNF114 [53]. Moreover, circPLCE1 interacts with HSC70, leading to an increase in its ubiquitination level in lung cancer [54], while circNF1 interacts with and blocks the deubiquitination of annexin A1 in esophageal squamous cell carcinoma [55]. Together, the findings of these studies demonstrate that dysregulated circRNA-mediated protein stability may represent a common mechanism underlying cancer progression.

Our study revealed a previously unidentified role of MYH9, a member of the myosin family, in the regulation of DNA repair. While it has been reported that MYO1A and MYO1B, also members of the myosin family, promote the movement of DSBs through their ability to walk along actin filaments [18, 56], our results showed that MYH9 enables the clustering of DSBs by promoting the organization of nuclear actin network. These results support that MYO1A, MYO1B and MYH9 have distinct functions in formation of functional actin filaments networks [57, 58]. While MYH9 is a conventional myosin, characterized by actin cross-linking and contractility properties, which self-associates into filaments and establishes cross-bridges with actin filaments, forming a functional actin-myosin network [25, 59, 60]. MYO1A and MYO1B are unconventional myosins, which anchor cargo on and move cargo along actin filaments [58, 61, 62]. Therefore, to achieve the movement of DSBs along nuclear actin filaments, MYO1A and MYO1B act as “molecular motors” [18], carrying DSB sites, and MYH9 is essential for organization of nuclear actin filaments that serve as tracks for such movement. Hence, our study underscores the important role of conventional myosin in the organization of nuclear actin filaments, which provide tracks for the trafficking of DSBs, thus enhancing the clustering of DSBs for efficient repair. Interestingly, the MYH9 mutation and its functional deficiency has been reported to cause abnormal platelet formation, and lead to a significant increase in the risk of patients developing hematological malignancies (especially AML and MPN) [63–65], indicating that MYH9 might be a potential therapeutic target for hemopoietic malignancy.

## Supplementary information

Supplemental Material

Table s1

Table s2

Table s3

Table s4

Supplementary Videos

Original western blots

## Data Availability

CircRNA expression profile data are available in the GEO databases (GSE292492). Raw circRNA-seq data of MDS patients from a previously published study can be accessed from the GSA database (PRJNA896500). The corresponding author provided all data and supplementary information within the article upon reasonable request.
