# Supplementary material for: Cancer-associated SF3B1 mutation suppresses DNA repair by disrupting the organization of nuclear actin network
Source: Cell Death Dis. 2026 Mar 21;17(1):334. doi: 10.1038/s41419-026-08569-5 (PMC13039298; doi:10.1038/s41419-026-08569-5)
Supplement: Supplementary file 1 — Supplemental Material [file 41419_2026_8569_MOESM1_ESM.docx]

**Supplementary information**

**Cancer-associated SF3B1 mutation suppresses DNA repair by disrupting the organization of nuclear actin network**

Rui Qian,^1^ Zhipeng Zhao,^2^ Xuanxuan Sun,^1^ Benkai Xin,^1^ Peipei An,^1^ Ting Yang,^1^ Ning Wu,^2^ Xin Hu^1,3^ and Youzhong Wan^1, *^

^1^Cancer Biology Laboratory, China-Japan Union Hospital of Jilin University, Jilin University, Changchun, Jilin, China.

^2^Department of Radiation Oncology, China-Japan Union Hospital of Jilin University, Jilin University, Changchun, Jilin, China.

^3^Department of Cardiology, China-Japan Union Hospital of Jilin University, Jilin University, Changchun, Jilin, China.

^*^ Lead Contact

Corresponding author Email: [wanyouzhong@jlu.edu.cn](mailto:wanyouzhong@jlu.edu.cn)

Present address: 126 Xiantai Street, Changchun, Jilin, China.

**This PDF file includes:**

Figures S1 to S13

Video legends S1 to S4

Tables S1 to S6

**Supplementary Figures**


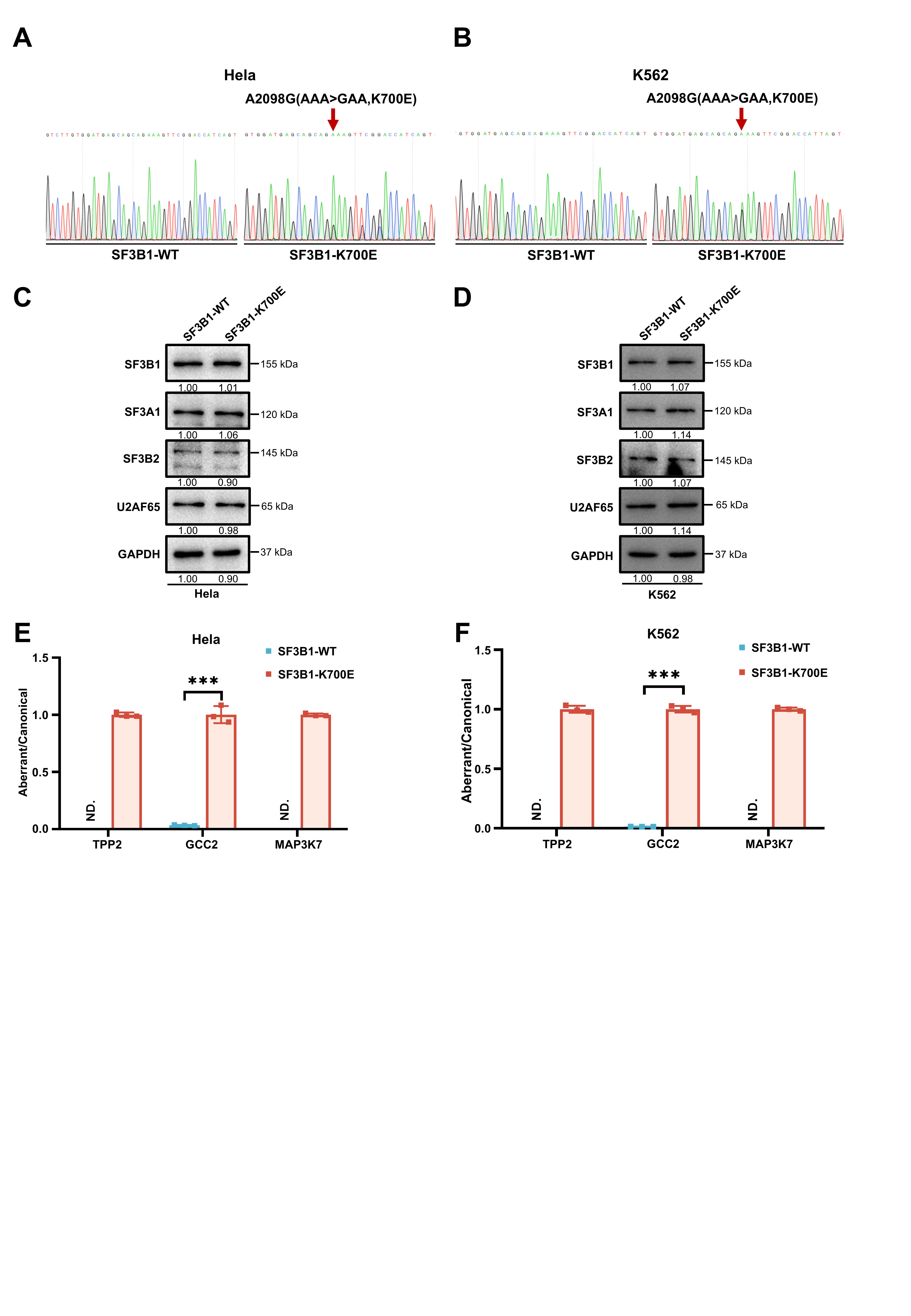


**Supplementary Figure 1. Characterization of SF3B1-K700E mutated cells generated by CRISPR/Cas 9.**

(A, B) Sanger sequencing of the targeted genomic region in Hela (A) and K562 (B) cells harboring SF3B1-K700E mutation.

(C, D) Western blots to show the expression of RNA splicing factors in Hela (C) and K562 (D) cells with or without SF3B1-K700E mutation using SF3B1, SF3A1, SF3B2, and U2AF65 antibody. GAPDH was used as control.

(E, F) Taqman qPCR analysis to show the alternative splicing events associated with SF3B1-K700E mutation in Hela (E) and K562 (F) cells. Data are expressed as the mean ± SD, one-way ANOVA with multiple comparisons (E and F). ND represents not detected, *** *p* < 0.001.


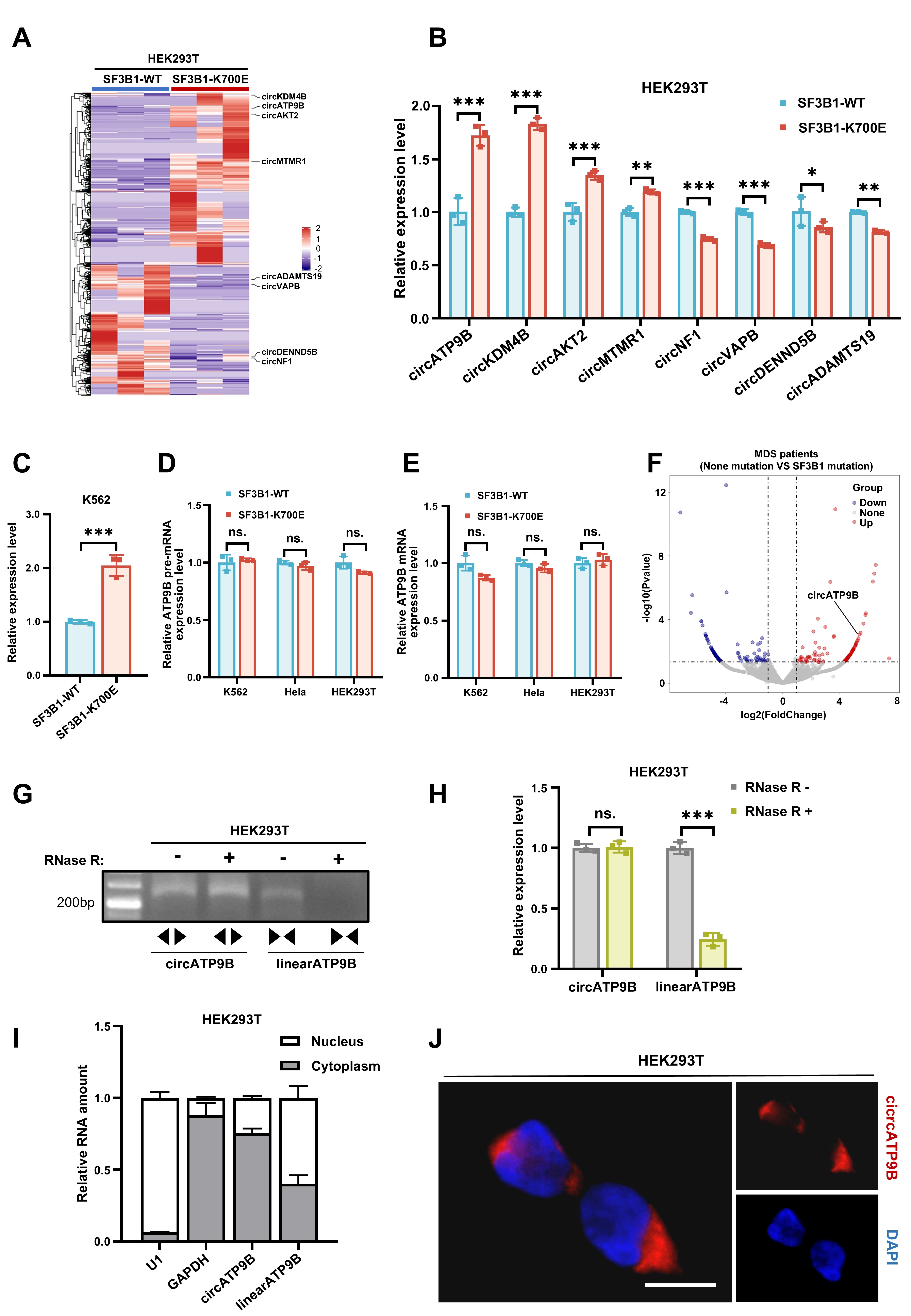


**Supplementary Figure 2. Identification and characterization of circATP9B in HEK293T and K562 cells with SF3B1 mutation.**

(A) Heatmap of the differentially expressed circRNAs in HEK293T cells transfected with wildtype (SF3B1-WT) or K700E mutated (SF3B1-K700E) SF3B1 expression constructs (*n* = 3 independent samples).

(B) qRT-PCR validation of the expression of some circRNAs in SF3B1-WT and SF3B1-K700E HEK293T cells.

(C) qRT-PCR analysis to show the expression of circATP9B in K562 cells with or without SF3B1-K700E mutation.

(D) qRT-PCR analysis to show the expression of ATP9B pre-mRNA in K562, Hela and HEK293T cells with or without SF3B1-K700E mutation.

(E) qRT-PCR analysis to show the expression of ATP9B mRNA in K562, Hela and HEK293T cells with or without SF3B1-K700E mutation.

(F) Volcano plot of the differentially expressed circRNAs in CD34+ BM cells from myelodysplastic syndrome (MDS) patients with or without SF3B1 mutation.

(G) Agarose gel electrophoresis of the PCR products amplified from total RNA of HEK293T cells that were treated or non-treated with RNase R, using divergent primers for circATP9B and convergent primers for linearATP9B.

(H) qRT-PCR analysis of the relative expression of circATP9B and linearATP9B in total RNA of HEK293T cells that were treated or non-treated with RNase R.

(I) Nuclear and cytoplasmic fractions of HEK293T cells were separated, and relative amount of circATP9B and linearATP9B in nuclear and cytoplasmic RNA was analyzed by qRT-PCR. U1 and GAPDH were served as nuclear and cytoplasmic markers, respectively.

(J) RNA fluorescence *in* *situ* hybridization assay to show the subcellular localization of circATP9B (red) in HEK293T cells. Nuclei were stained with DAPI (blue), scale bar = 10 μm. Data are expressed as the mean ± SD, one-way ANOVA with multiple comparisons (B, C, D, E, H and I). ns represents not significant, * *p* < 0.05, ** *p* < 0.01, and *** *p* < 0.001.


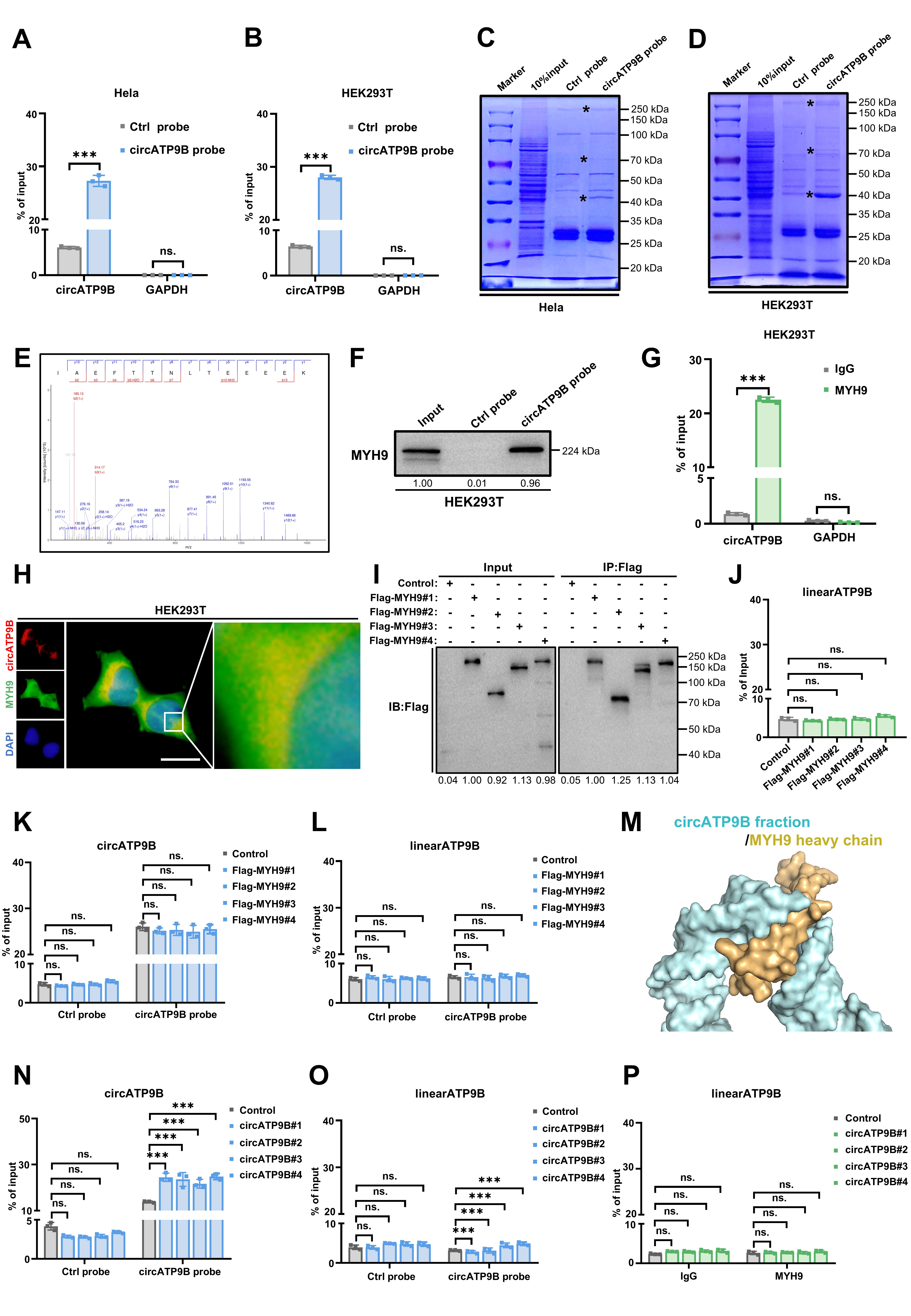


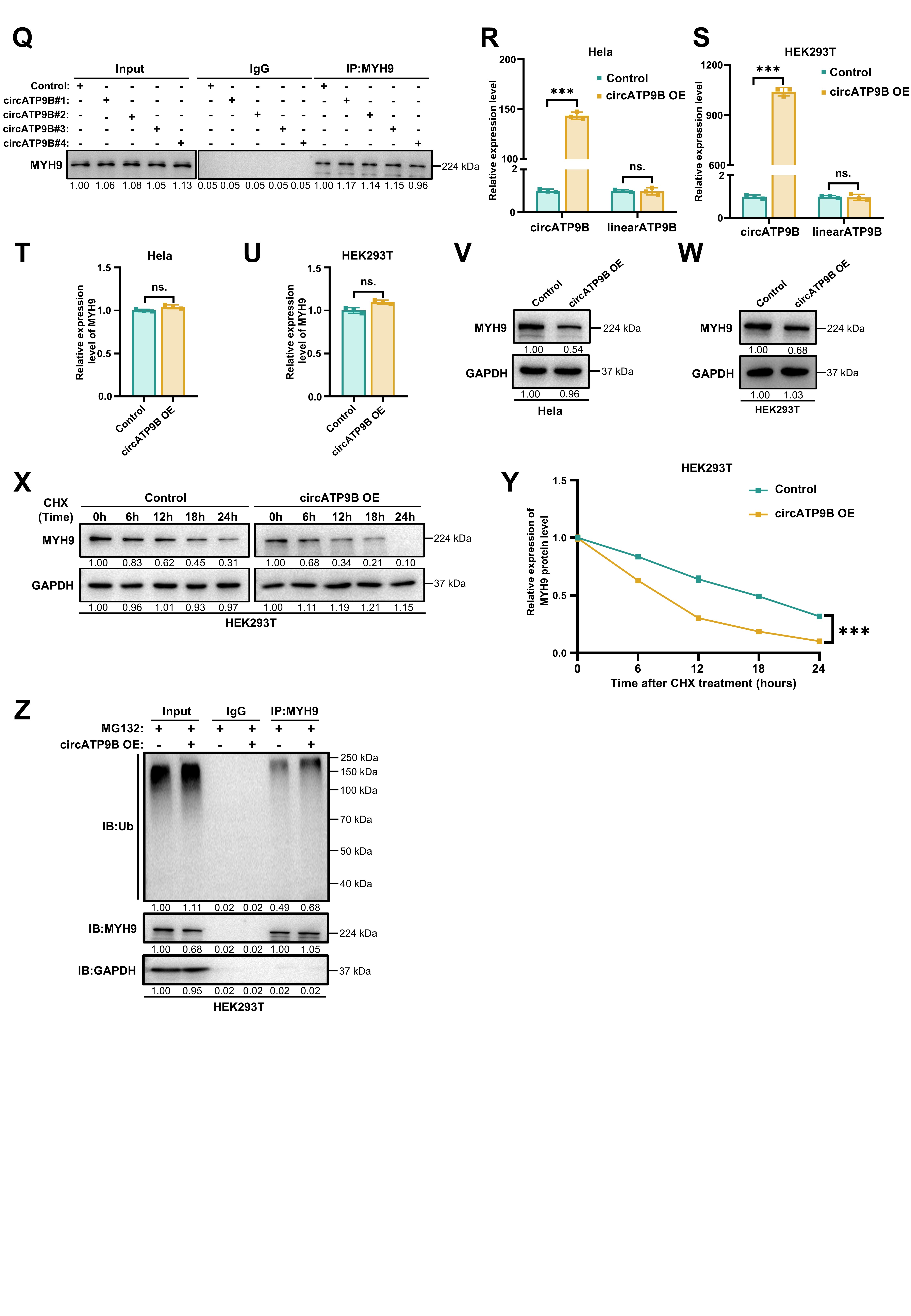


**Supplementary Figure 3. CircATP9B interacts with MYH9 in HEK293T cells.**

(A, B) RNA pulldown and qRT-PCR analysis of the enrichment of circATP9B using the circATP9B probe and control probe (Ctrl probe) in Hela (A) and HEK293T (B) cells.

(C, D) Analysis of circATP9B binding proteins in Hela (C) and HEK293T(D) cells using sodium dodecyl-sulfate polyacrylamide gel electrophoresis. The gels were stained with Coomassie brilliant blue R250. The bands showing proteins that were specifically pulled down by circATP9B probe were marked by asterisks.

(E) Specific peptide fragment from MYH9 were identified by LC-MS/MS.

(F) Pulldown using circATP9B and Ctrl probe and western blot analysis to confirm the interaction between circATP9B and MYH9 in HEK293T cells.

(G) RNA immunoprecipitation analysis using MYH9 antibody and IgG as control to confirm the interaction between circATP9B and MYH9 in HEK293T cells. GAPDH was used as control.

(H) Representative immunofluorescence images of the localization of circATP9B (red) and MYH9 (green) in HEK293T cells. Nuclei were stained with DAPI (blue), scale bar = 10 μm.

(I, J) RIP assays with anti-Flag antibody in HEK293T cells transfected with MYH9 constructs. Co-precipitated proteins and RNAs were analyzed by western blot (I) and qRT-PCR (J), respectively.

(K, L) RNA pull-down assays using biotin-labeled circATP9B probe and Ctrl probe in HEK293T cells expressing full-length or truncated MYH9, and subjected to qRT-PCR of circATP9B (K) and linearATP9B (L).

(M) circATP9B and MYH9 docking model was generated by HDOCK.

(N, O) RNA pull-down assays using biotin-labeled Ctrl probe and circATP9B probe in HEK293T cells transfected with indicated circATP9B expression constructs. Co-precipitated RNAs were analyzed by qRT-PCR of circATP9B (N) and llinearATP9B (O).

(P, Q) RIP assays with anti-MYH9 antibody in HEK293T cells transfected with indicated circATP9B expression constructs. Co-precipitated proteins and RNAs were analyzed by qRT-PCR (P) and western blot (Q), respectively.

(R, S) qRT-PCR analysis of the expression of circATP9B and linearATP9B in Hela (R) and HEK293T (S) cells transfected with circATP9B expression constructs.

(T, U) qRT-PCR analysis to show the expression of MYH9 RNA in Hela (T) and HEK293T (U) cells transfected with circATP9B expression constructs.

(V, W) Western blot to show the expression of MYH9 protein in control and circATP9B overexpression Hela (V) and HEK293T (W) cells. GAPDH was used as control.

(X) Western blots to show the expression of MYH9 in control and circATP9B overexpression HEK293T cells that were treated with 100 μM cycloheximide at the indicated times. GAPDH was used as control.

(Y) Quantification of the relative expression of MYH9 protein by calculating the intensities of western blot bands shown in (X).

(Z) Western blot to show ubiquitination level of MYH9 in control and circATP9B overexpression HEK293T cells treated with 20 nM MG132 for 12 h. Data are expressed as the mean ± SD (A, B, G, J-L, N-P, R-U and Y), unpaired two-tailed Student’s *t*-test (T and U), one-way ANOVA (A, B, G, J, K, L, N-P, R and S) and two-way ANOVA with multiple comparisons (M). ns represents not significant, and *** *p* < 0.001.


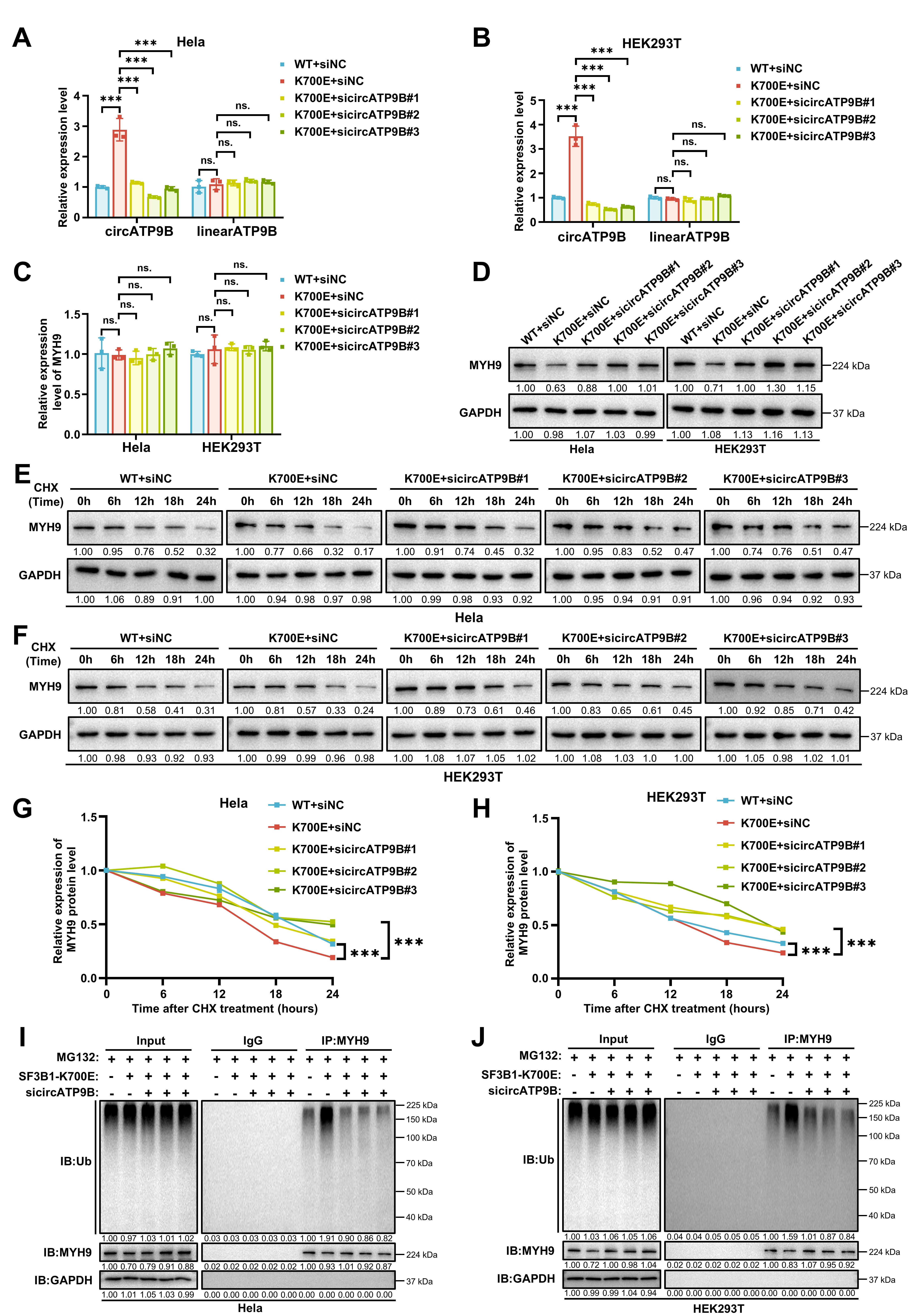


**Supplementary Figure 4. CircATP9B knockdown suppresses degradation of MYH9 protein in cells with SF3B1-K700E mutation.**

(A, B) qRT-PCR analysis to show the expression of circATP9B and linearATP9B in wildtype SF3B1 and control siRNA transfected (WT+siNC), SF3B1-K700E mutated and control siRNA transfected (K700E+siNC), SF3B1-K700E mutated and circATP9B siRNA transfected (K700E+sicircATP9B) Hela (A) and HEK293T (B) cells.

(C) qRT-PCR analysis to show the expression of MYH9 in WT+siNC, K700E+siNC, K700E+sicircATP9B Hela and HEK293T cells.

(D) Western blot to show the expression of MYH9 protein in WT+siNC, K700E+siNC, K700E+sicircATP9B Hela and HEK293T cells.

(E, F) Western blots to show the expression of MYH9 in WT+siNC, K700E+siNC, K700E+sicircATP9B Hela (E) and HEK293T (F) cells that were treated with cycloheximide at the indicated times. GAPDH was used as control.

(G, H) Quantification of the relative expression of MYH9 protein by calculating the intensities of western blot bands shown in (E) and (F), respectively.

(I, J) Western blot to show ubiquitination level of MYH9 in WT+siNC, K700E+siNC, K700E+sicircATP9B Hela (I) and HEK293T (J) cells treated with 20nm MG132 for 6 h. Data are expressed as the mean ± SD (A-C, G and H), one-way ANOVA (A-C) and two-way ANOVA with multiple comparisons (G and H). ns represents not significant, * *p* < 0.05, ** *p* < 0.01, and *** *p* < 0.001.


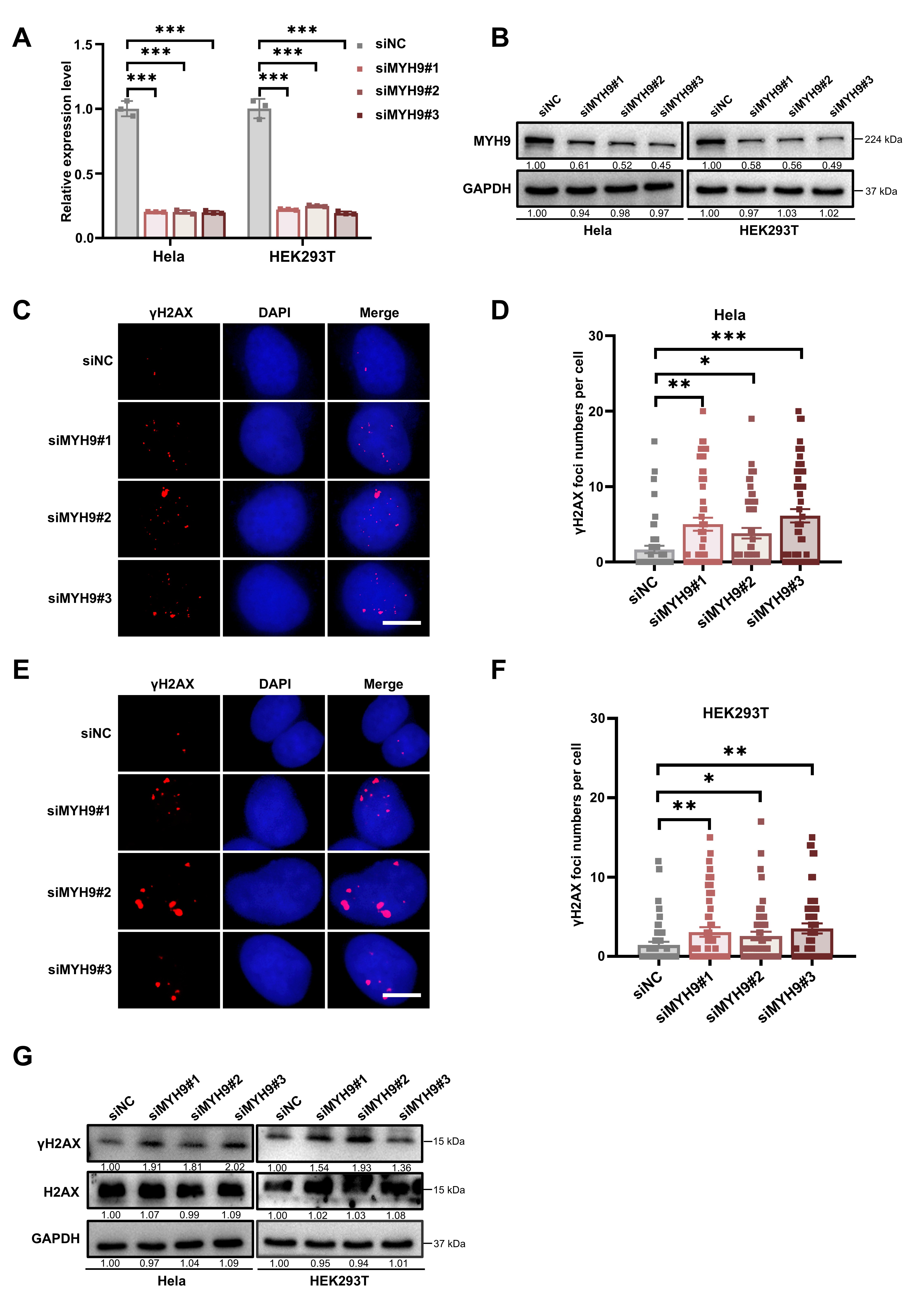


**Supplementary Figure 5. MYH9 knockdown enhances DNA damage.**

(A) qRT-PCR analysis to show the expression of MYH9 RNA in Hela and HEK293T cells transfected with control siRNA (siNC) or MYH9 siRNA (siMYH9).

(B) Western blot to show the expression of MYH9 protein in Hela and HEK293T cells transfected with siNC or siMYH9.

(C, E) Representative Immunofluorescence images of γH2AX foci in non-irradiated Hela (C) and HEK293T (E) cells transfected with siNC or siMYH9. γH2AX was stained with red, nuclei were stained with DAPI (blue), scale bar = 10 μm.

(D, F) Quantification of γH2AX foci in non-irradiated Hela (D) and HEK293T (F) cells transfected with siNC or siMYH9 (50 cells per group).

(G) Western blot to show the expression of γH2AX in Hela and HEK293T cells transfected with siNC or siMYH9. H2AX and GAPDH was used as control. Data are expressed as the mean ± SD (A) or SEM (D and F), one-way ANOVA with multiple comparisons (A, D and F). * *p* < 0.05, ** *p* < 0.01, and *** *p* < 0.001.


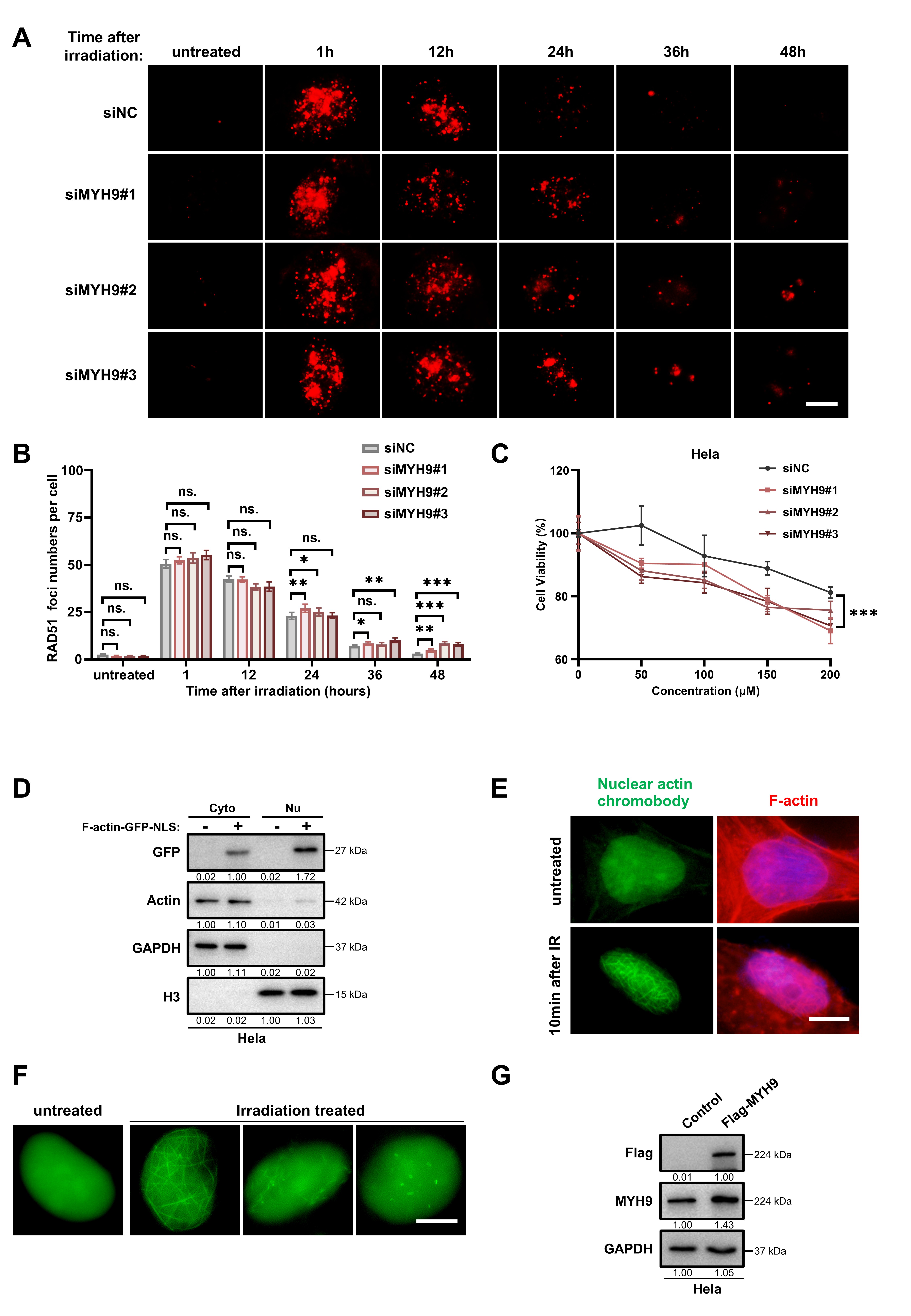


**Supplementary Figure 6. MYH9 knockdown reduces the efficiency of homology-directed repair.**

(A) Representative immunofluorescence images of RAD51 foci in MYH9 knockdown Hela cells at different times after irradiation. RAD51 was stained with red, scale bar = 10 μm.

(B) Quantification of RAD51 foci as shown in (A) after irradiation (50 cells per group).

(C) Cell Counting Kit-8 assay for the viability of MYH9 knockdown Hela cells treated with different doses of olaparib.

(D) Western blot to show the expression of actin and GFP in cytoplasmic and nuclear fractions of Hela cells. GAPDH and H3 served as cytoplasmic and nuclear markers, respectively.

(E) Representative immunofluorescence images of nuclear actin filaments after irradiation. F-actin was visualized with iFlour 555-phalloidin label (red), nuclei were stained with DAPI (blue), scale bar = 10 μm.

(F) Representative images of the differences in the morphology of nuclear actin after irradiation treatment. Scale bar = 10 μm.

(G) Western blot to show the expression of Flag and MYH9 in Hela cells transfected with Flag-MYH9 plasmid. GAPDH was used as control. Data are expressed as the mean ± SEM (B) or SD (C), unpaired two-tailed Student’s *t*-test (B) and two-way ANOVA with multiple comparisons (C). ns represents not significant, * *p* < 0.05, ** *p* < 0.01, and *** *p* < 0.001.


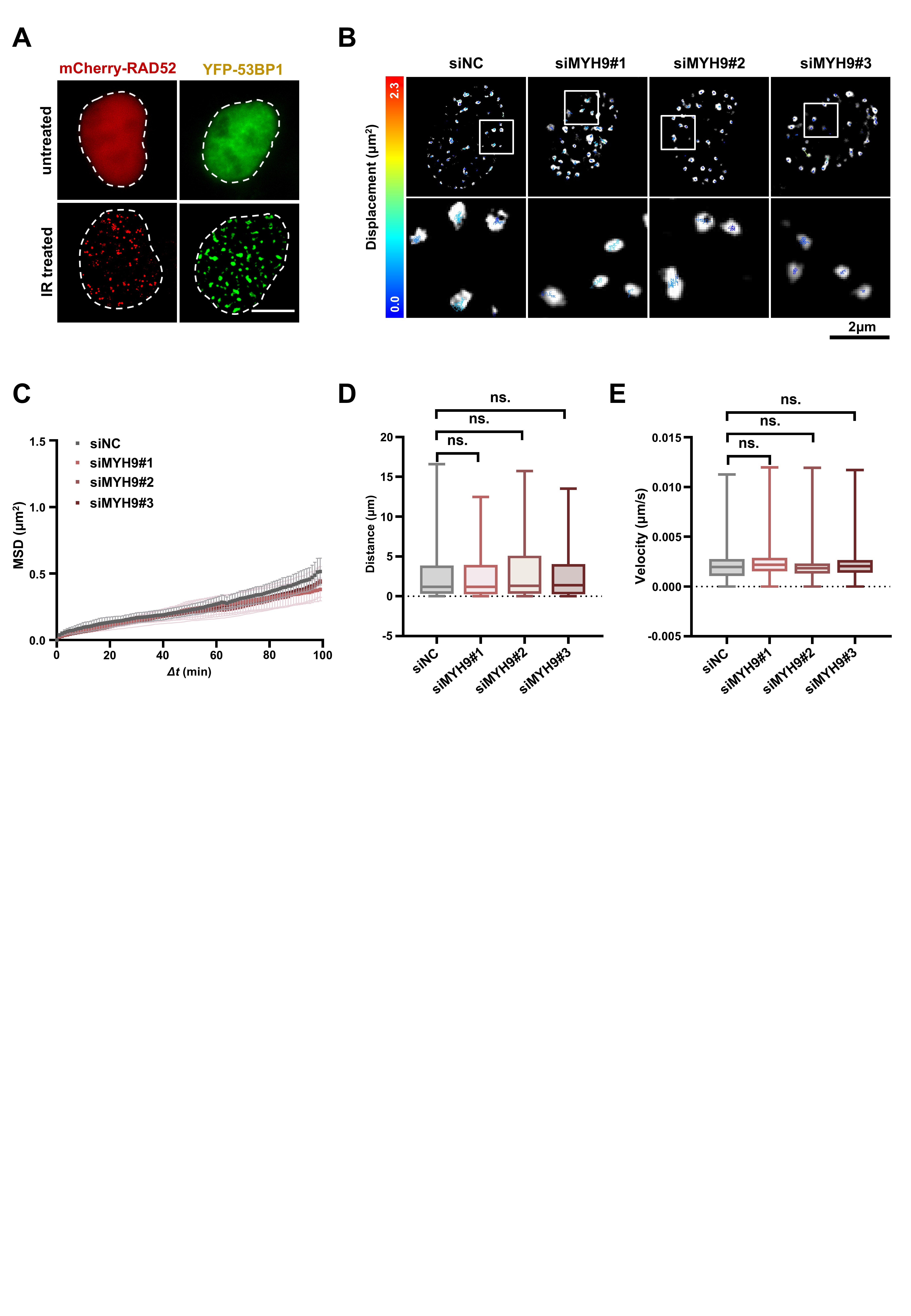


**Supplementary Figure 7. MYH9 knockdown does not affect the mobility of YFP-53BP1 foci.**

(A) Images of the automated aggregation of mCherry-RAD52 and YFP-53BP1 foci in Hela cells after irradiation. Scale bar = 10 μm.

(B) Representative images of YFP-53BP1 foci traces in Hela cells transfected with siNC or siMYH9 over 100 min after irradiation. Scale bar = 2 μm.

(C-E) MSD (C), distance (D), velocity (E) of YFP-53BP1 foci in Hela cells transfected with siNC or siMYH9. 1173 foci from 10 nuclei of siNC, 784 foci from 10 nuclei of siMYH9#1, 830 foci from 10 nuclei of siMYH9#2, 733 foci from 10 nuclei of siMYH9#3. *Δt*, time intervals. Data are expressed as the mean ± SEM (C-E), two-way ANOVA (C) and one-way ANOVA with multiple comparisons (D and E). ns represents not significant.


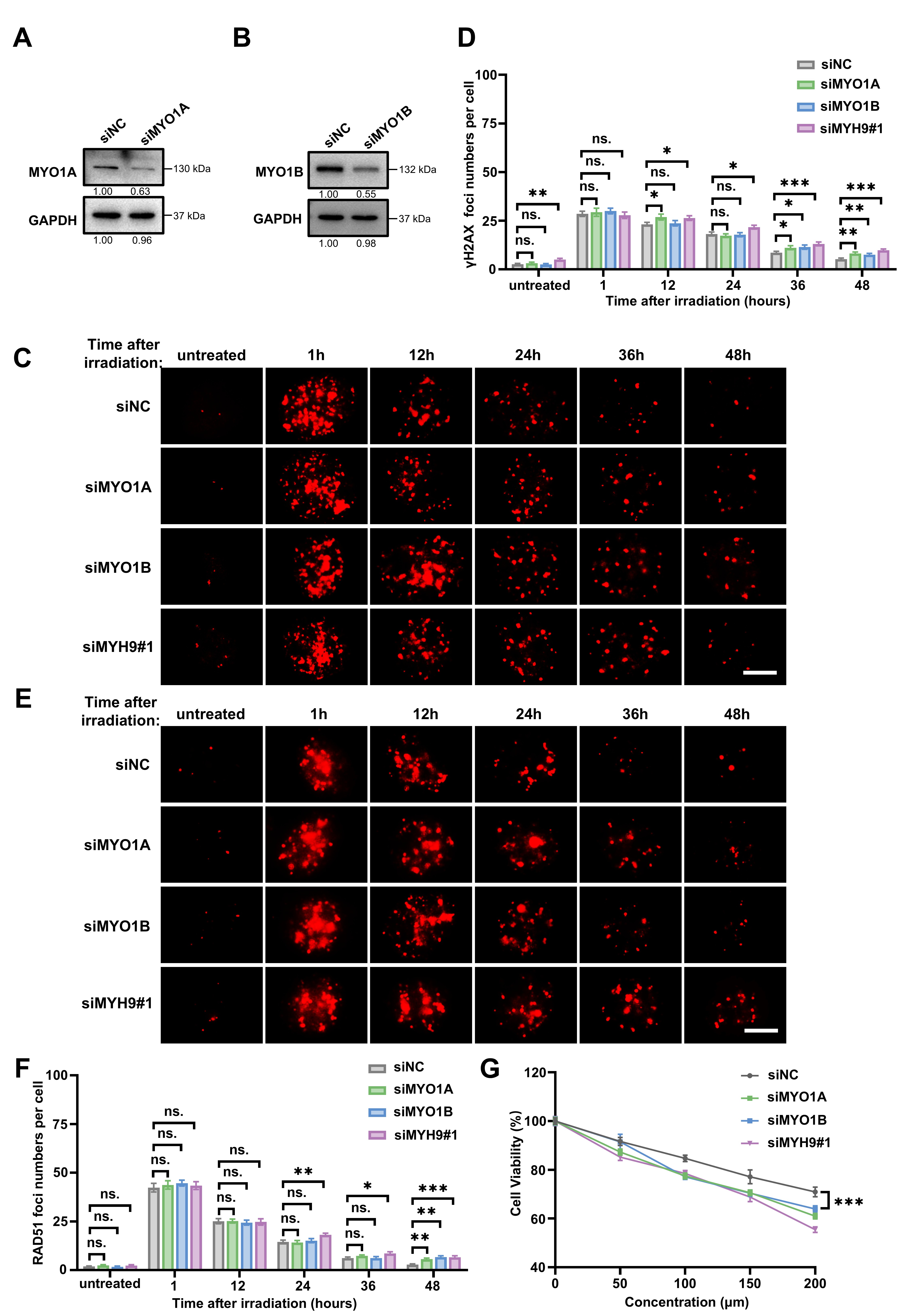


**Supplementary Figure 8. Knockdown of MYO1A and MYO1B inhibits DNA repair.**

(A) Western blot to show the expression of MYO1A protein in Hela cells transfected with control siRNA (siNC) or MYO1A siRNA (siMYO1A).

(B) Western blot to show the expression of MYO1B protein in Hela cells transfected with control siRNA (siNC) or MYO1B siRNA (siMYO1B).

(C) Representative immunofluorescence images of γH2AX foci in Hela cells transfected with the indicated siRNA at different times after irradiation. γH2AX was stained with red, scale bar = 10 μm.

(D) Quantification of the number of γH2AX foci as shown in (C) (50 cells per group).

(E) Representative immunofluorescence images of RAD51 foci in Hela cells transfected with the indicated siRNA at different times after irradiation. RAD51 was stained with red, scale bar = 10 μm.

(F) Quantification of the number of RAD51 foci as shown in (E) (50 cells per group).

(G) Cell Counting Kit-8 assay for the viability of Hela cells transfected with the indicated siRNA and then treated with different doses of olaparib. Data are expressed as the mean ± SEM (D and F) and SD (G), unpaired two-tailed Student’s *t*-test (D and F) and two-way ANOVA with multiple comparisons (G). ns represents not significant, * *p* < 0.05, ** *p* < 0.01, and *** *p* < 0.001.


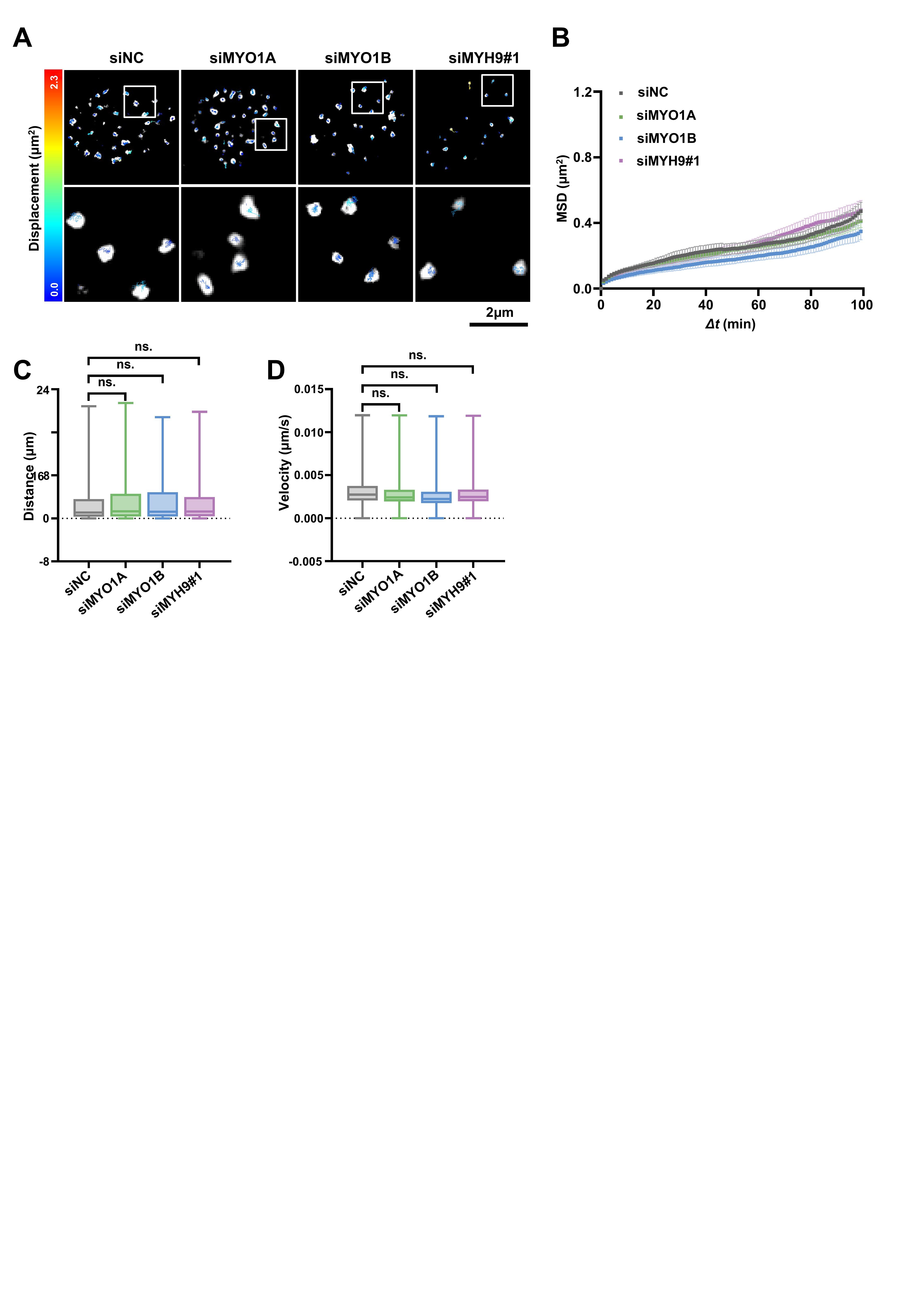


**Supplementary Figure 9. Knockdown of MYO1A, MYO1B, and MYH9 do not affect the movement of YFP-53BP1 foci.**

(A) Representative images of YFP-53BP1 foci traces in Hela cells transfected with the indicated siRNA over 100 min after irradiation. Scale bar = 2 μm.

(B-D) MSD (B), distance (C), velocity (D) of YFP-53BP1 foci in Hela cells transfected with the indicated siRNA. 1713 foci from 13 nuclei of siNC,1408 foci from 15 nuclei of siMYO1A, 781 foci from 15 nuclei of siMYO1B, 1041 foci from 14 nuclei of siMYH9#1. *Δt*, time intervals. Data are expressed as the mean ± SEM (B-D), two-way ANOVA (B) and one-way ANOVA with multiple comparisons (C and D). ns represents not significant.


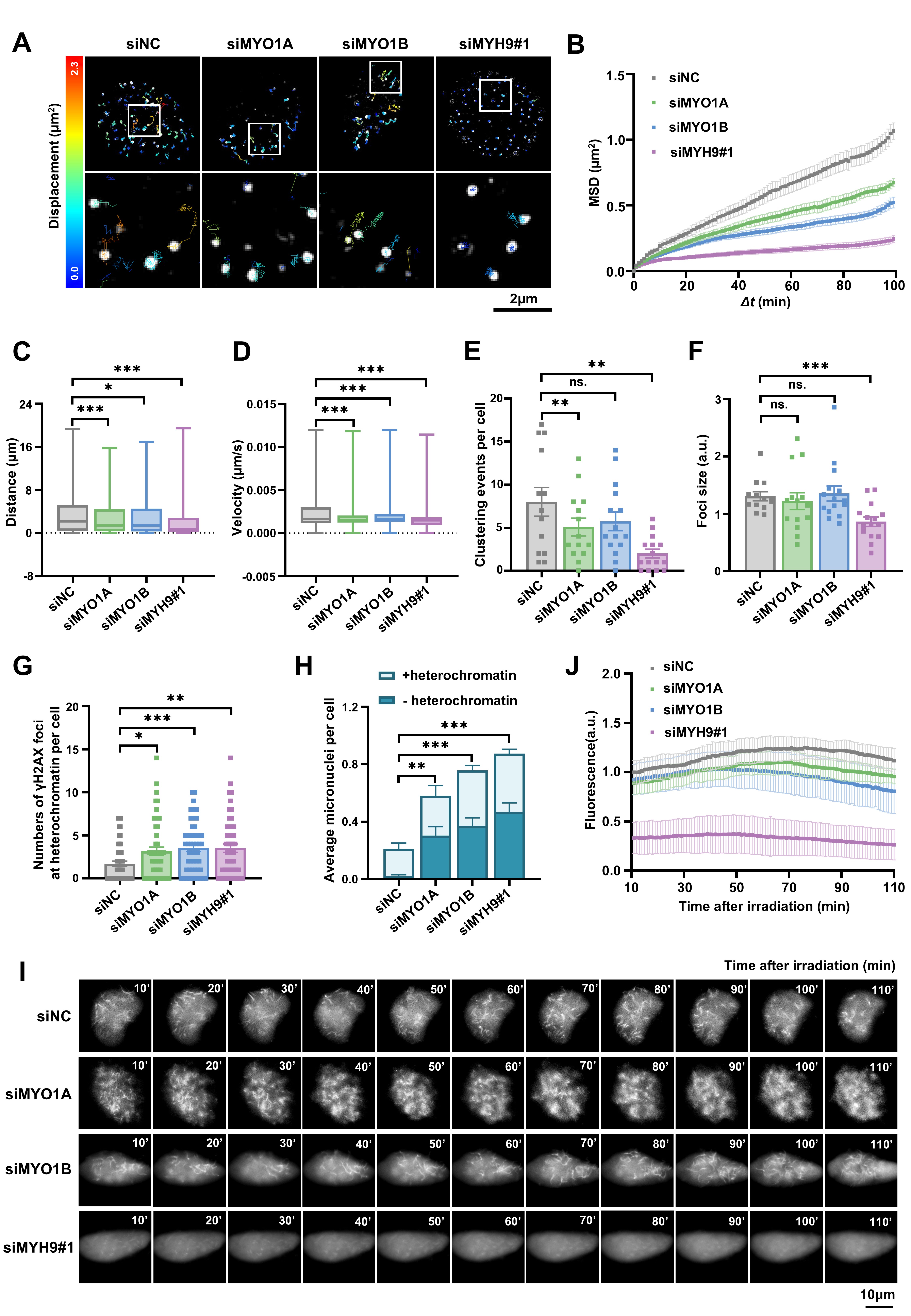


**Supplementary Figure 10. Knockdown of MYO1A and MYO1B inhibit the mobility of DSB sites but do not affect organization of nuclear actin network**.

(A) Representative images of mCherry-RAD52 foci traces in Hela cells transfected with the indicated siRNA over 100 min after irradiation. Scale bar = 2 μm.

(B-F) MSD (B), distance (C), velocity (D), sizes (E), clustering events (F) of mCherry-RAD52 foci in Hela cells transfected with the indicated siRNA. 1659 foci from 13 nuclei of siNC,1543 foci from 14 nuclei of siMYO1A, 1755 foci from 15 nuclei of siMYO1B, 1459 foci from 15 nuclei of siMYH9#1. *Δt*, time intervals.

(G, H) Numbers of γH2AX foci (G) and micronuclei associated with HP1α (H) in Hela cells transfected with the indicated siRNA at 48h after irradiation (50 cells per group for γH2AX foci, *n* > 120 cells per group for micronuclei).

(I) Representative images of nuclear actin filaments in Hela cells transfected with the indicated siRNA and subjected to irradiation. Scale bar =10 μm.

(J) Quantification of fluorescence intensities from nuclear actin filaments as shown in (I) (*n* ≥ 15). Data are expressed as the mean ± SEM (B-H) and SD (J), one-way ANOVA (C-H) and two-way ANOVA with multiple comparisons (B and J). ns represents not significant, * *p* < 0.05, ** *p* < 0.01, and *** *p* < 0.001.


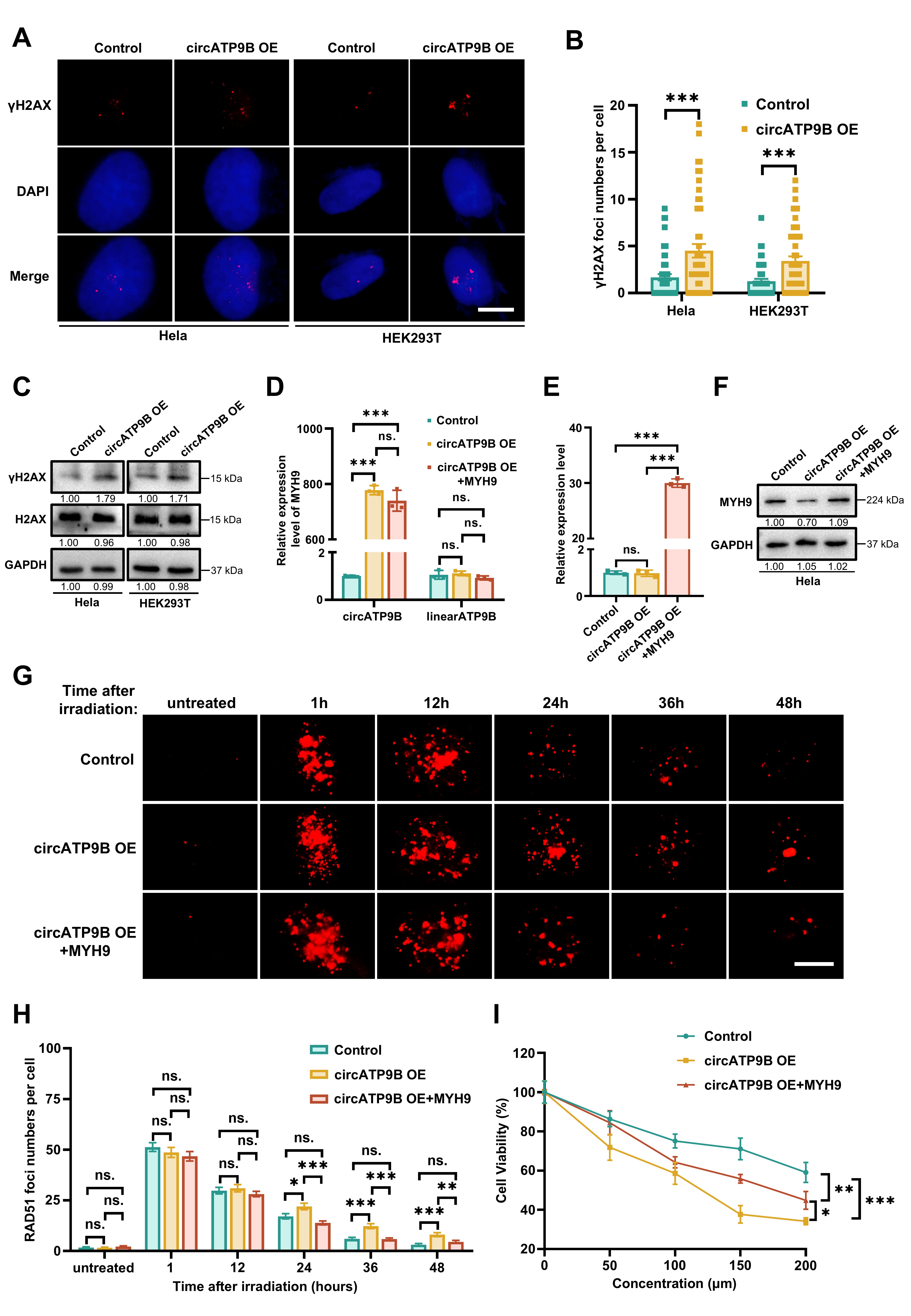


**Supplementary Figure 11. MYH9 overexpression rescues the decreased efficiency of cells with circATP9B overexpression.**

(A) Representative immunofluorescence images of γH2AX foci in Hela and HEK293T cells with circATP9B overexpression. γH2AX was stained with red, nuclei were stained with DAPI (blue), scale bar = 10 μm.

(B) Quantification of γH2AX foci in Hela and HEK293T cells with circATP9B overexpression as shown in (A) (50 cells per group).

(C) Western blot to show the expression of γH2AX in Hela and HEK293T cells with circATP9B overexpression. GAPDH and H2AX was used as control.

(D) qRT-PCR analysis to show the expression of circATP9B and linearATP9B in control, circATP9B overexpression (circATP9B OE), circATP9B and MYH9 overexpression (circATP9B OE+MYH9) Hela cells.

(E) qRT-PCR analysis to show the expression of MYH9 RNA in control, circATP9B OE, circATP9B and circATP9B OE+MYH9 Hela cells.

(F) Western blot to show the expression of MYH9 protein in control, circATP9B OE, circATP9B and circATP9B OE+MYH9 Hela cells. GAPDH was used as control.

(G) Representative immunofluorescence images of RAD51 foci in control, circATP9B OE, circATP9B OE+MYH9 Hela cells at different times after irradiation. RAD51 was stained with red, scale bar = 10 μm.

(H) Quantification of RAD51 foci as shown in (F) after irradiation (50 cells per group).

(I) Cell Counting Kit-8 assay for the viability of control, circATP9B OE, circATP9B OE+MYH9 Hela cells and treated with different concentrations of olaparib. Data are expressed as the mean ± SEM (B and H) or SD (D, E, I), unpaired two-tailed Student’s *t*-test (H), one-way ANOVA (B, D, E) and two-way ANOVA with multiple comparisons (I). ns represents not significant, * *p* < 0.05, ** *p* < 0.01, and *** *p* < 0.001.


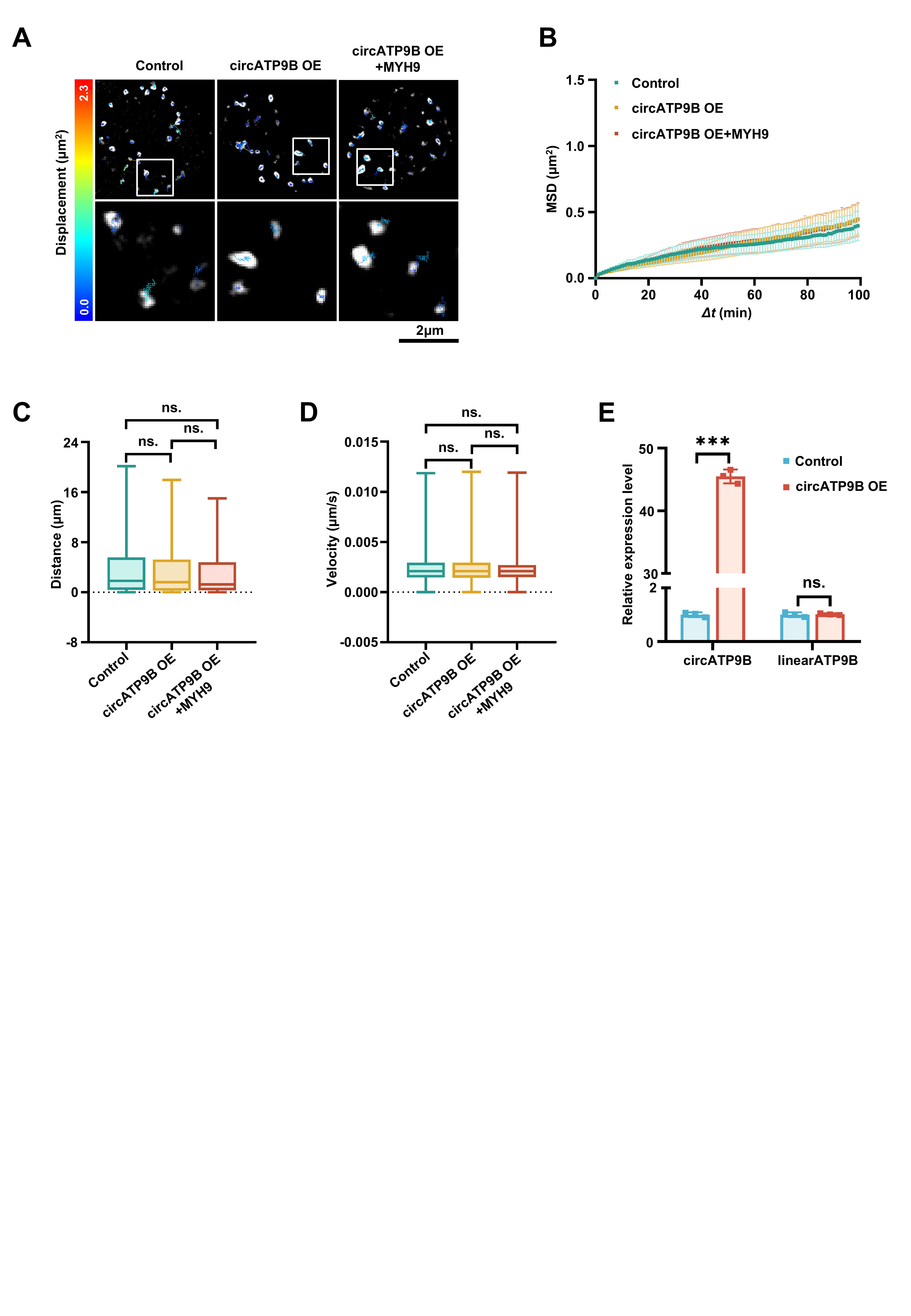


**Supplementary Figure 12. CircATP9B overexpression does not affect the mobility of YFP-53BP1 foci.**

(A) Representative images of YFP-53BP1 foci traces in control, circATP9B OE, circATP9B OE+MYH9 Hela cells over 100 min after irradiation. Scale bar = 2 μm.

(B-D) MSD (B), distance (C), velocity (D) of YFP-53BP1 foci in control, circATP9B OE, circATP9B OE+MYH9 Hela cells. 1145 foci from 10 nuclei of control,744 foci from 10 nuclei of circATP9B OE, 581 foci from 10 nuclei of circATP9B OE+MYH9. *Δt*, time intervals.

(E) qRT-PCR analysis to show the expression of MYH9 RNA in control and circATP9B OE Hela cells used in xenograft. Data shown as mean ± SEM (B-D) and ± SD (E), two-way ANOVA with multiple comparisons (B), one-way ANOVA with multiple comparisons (C-E). ns represents not significant, and *** *p* < 0.001.


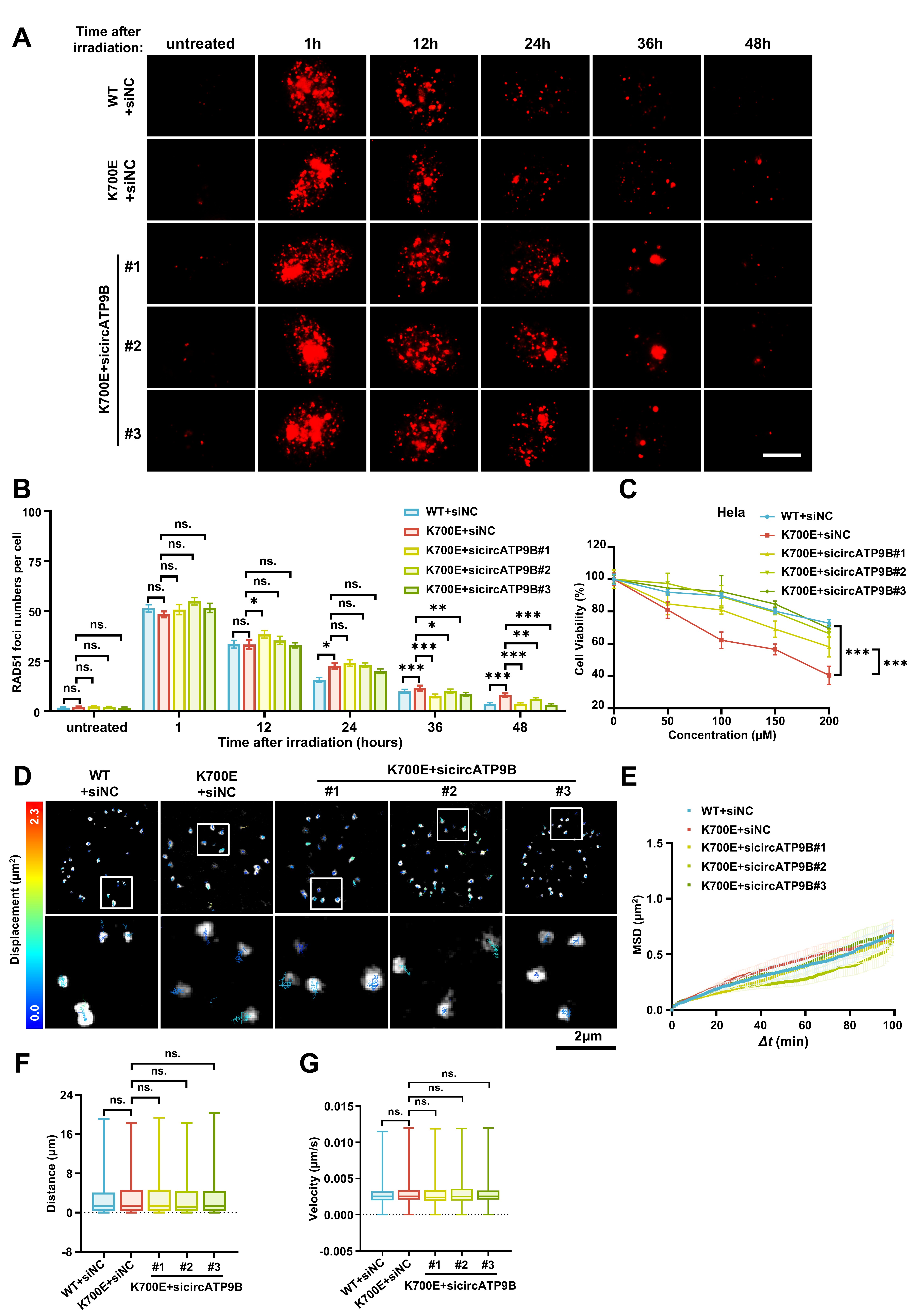


**Supplementary Figure 13. circATP9B knockdown improves the efficiency of homology-directed repair in Hela cells with SF3B1-K700E mutation.**

(A) Representative immunofluorescence images of RAD51 foci in WT+siNC, K700E+siNC, K700E+sicircATP9B Hela cells at indicated times after irradiation. RAD51 was stained with red, scale bar = 10 μm.

(B) Quantification of RAD51 foci as shown in (A) (50 cells per group).

(C) Cell Counting Kit-8 assay for the viability of WT+siNC, K700E+siNC, K700E+sicircATP9B Hela cells treated with different concentrations of olaparib.

(D) Representative images of YFP-53BP1 foci traces in WT+siNC, K700E+siNC, K700E+sicircATP9B Hela cells over 100 min after irradiation. Scale bar = 2 μm.

(E-G) MSD (E), distance (F), velocity (G) of YFP-53BP1 foci in WT+siNC, K700E+siNC, K700E+sicircATP9B Hela cells. 694 foci from 10 nuclei of WT+siNC,1015 foci from 10 nuclei of K700E+siNC, 991 foci from 10 nuclei of K700E+sicircATP9B#1,782 foci from 10 nuclei of K700E+sicircATP9B#2, 1572 foci from 10 nuclei of K700E+sicircATP9B#3. *Δt*, time intervals. Data are expressed as the mean ± SEM (B, E-G) or SD (C), unpaired two-tailed Student’s *t*-test (B). one-way ANOVA (F and G) and two-way ANOVA with multiple comparisons (C and E). ns represents not significant, * *p* < 0.05, ** *p* < 0.01, and *** *p* < 0.001.

**Supplementary video legends**

**Supplementary video 1. MYH9 mediates organization of nuclear actin filaments and the movement of DSB sites during DNA repair.**

(A–D) Representative videos of nuclear actin filament in Hela cells transfected with control siRNA (siNC) or MYH9 siRNA (siMYH9) over 100 min after irradiation. Images are acquired every 1 min. Each frame in the videos is 10 min.

(E–H) Representative videos of mCherry-RAD52 foci over 100 min in Hela cells transfected with siNC or siMYH9 over 100 min after irradiation. Images are acquired every 1 min. Each frame in the videos is 10 min.

(I–L) Representative videos of YFP-53BP1 foci in Hela cells transfected with siNC or siMYH9 over 100 min after irradiation. Images are acquired every 1 min. Each frame in the videos is 10 min.

**Supplementary video 2 Knockdown of MYO1A/MYO1B inhibits the movement of DSB sites but not the organization of nuclear actin network.**

(A-D) Representative videos of YFP-53BP1 foci in Hela cells transfected with indicated siRNA over 100 min after irradiation, respectively. Images are acquired every 1 min. Each frame in the videos is 10 min.

(E-H) Representative videos of mCherry-RAD52 foci in Hela cells transfected with indicated siRNA over 100 min after irradiation, respectively. Images are acquired every 1 min. Each frame in the videos is 10 min.

(I-L) Representative videos of nuclear actin filaments in Hela cells transfected with indicated siRNA over 100 min after irradiation, respectively. Images are acquired every 1 min. Each frame in the videos is 10 min.

**Supplementary video 3 MYH9 overexpression enhances the organization of nuclear actin filaments and the mobility of DSB sites in Hela cells with circATP9B overexpression.**

(A-C) Representative videos of nuclear actin filaments in control, circATP9B OE, circATP9B OE+MYH9 Hela cells over 100 min after irradiation. Images are acquired every 1 min. Each frame in the videos is 10 min.

(D-F) Representative videos of mCherry-RAD52 foci in control, circATP9B OE, circATP9B OE+MYH9 Hela cells over 100 min after irradiation. Images are acquired every 1 min. Each frame in the videos is 10 min.

(G-I) Representative videos of YFP-53BP1 foci in control, circATP9B OE, circATP9B OE+MYH9 Hela cells over 100 min after irradiation. Images are acquired every 1 min. Each frame in the videos is 10 min.

**Supplementary video 4 circATP9B knockdown enhances the organization of nuclear actin filaments and the mobility of DSB sites in Hela cells with SF3B1-K700E mutation.**

(A-E) Representative videos of nuclear actin filaments in WT+siNC, K700E+siNC, K700E+sicircATP9B Hela cells over 100 min after irradiation. Images are acquired every 1 min. Each frame in the videos is 10 min.

(F-J) Representative videos of mCherry-RAD52 foci in WT+siNC, K700E+siNC, K700E+sicircATP9B Hela cells over 100 min after irradiation. Images are acquired every 1 min. Each frame in the videos is 10 min.

(K-O) Representative videos of YFP-53BP1 foci in WT+siNC, K700E+siNC, K700E+sicircATP9B Hela cells over 100 min after irradiation. Images are acquired every 1 min. Each frame in the videos is 10 min.

**Supplementary Tables**

| **Primer name** | **Forward (5’-3’)** | **Reverse (5’-3’)** |
| --- | --- | --- |
| circATP9B | TCGAAGGCACATTTACCAGG | TCCTTGTCACGCTGAAAACGC |
| circZEB1 | GAAAGTGTGGAAGCAAAGGTC | TGTGTCAAGCAGACAGTAGCC |
| circPTPRM | TCAGCACTCTCTATGCCAGC | GACGCCTTGTATGGATTCAG |
| circSCAMP1 | GCGGTTGATTTTGGATTGAG | GGTGTCATTATTGCTGGTTGTG |
| circEMB | GCAGGAGCTTCATCAACGAT | TCAAATCCCCAGATGTTGTG |
| circBIRC6 | CAGACAGTGAAGAGCATTCCAG | TCCTTCCTACACCCTACTGC |
| circTSHZ2 | GCAAGTCACCCGAACACCAT | GTTGAGATACTGAACCGCTG |
| circHIBADH | ACTGTGCAATGACCGTGAAC | GTCTTTCTGCTCCTTAGTCC |
| circRIMS1 | GTGTCTCGCAGGAGTAGAAGCA | CACTTTCATTTTCATCTGTCTTG |
| circKDM4B | CTGGCCAACAGCGAGAAGTA | GGATTTGTCAGGTGCCTCCA |
| circAKT2 | CCGCTACTACGCCATGAAGAT | CCTCAGTCGTGGAGGAGTCA |
| circMTMR1 | CTGGCTATGCTAATGTTGGA | AGTTCTGGATTTGGGTAAGC |
| circNF1 | TCTCTCAGCTGCAACAACTTCA | ATGTGTGTTCTGCTGTCCTGT |
| circVAPB | GGCTACGGGAGGAGAACAAG | TCTGTCTTTGATGCAGTTGTGG |
| circDENND5B | CTGGACAACGACTTGAGGGA | TCCGAGTGTCAAAGACCCGA |
| circADAMTS19 | TGACTTCAGTGGATGCAGCTAT | GGAAACCATTGCTGGGTCTG |
| ATP9B mRNA | TTGTCGCCAAGCACCATT | TTCGTCAGCCGATTGAGT |
| ATP9B pre-mRNA | TGAAAGCTCGCACAGTAT | GTCTCAACAGGCTCCATC |
| β-actin | GCCGCCAGCTCACCAT | TCGATGGGGTACTTCAGGGT |
| GAPDH | AGGGCTGCTTTTAACTCTGGT | CCCCACTTGATTTTGGAGGGA |
| U1 | GGGAGATAACGTGACCACGAAG | CCACAAATTATGCAGTCGAGTTTC |
| MYH9 | CAGCAAGCTGCCGATAAGTAT | CTTGTCGGAAGGCACCCAT |

**Table S5 qRT-PCR primer sequences.**

| **Primer name** | **Forward (5’-3’)** | **Reverse (5’-3’)** |
| --- | --- | --- |
| circATP9B | GCTGAAGGTGGCAGTGAG | AACAGGCAAGACAAATCC |
| linearATP9B | CATTTACCAGGGAAGACA | GCTCAAGGTCCAACAAAC |

**Table S6 PCR primer sequences.**

**Table S7 Taqman qPCR primer sequences.**

| **Name** | **Sequence** |
| --- | --- |
| TPP2 Canonical Forward | CTCCCTGCATAACTTTGAAGAACTG |
| TPP2 Canonical Reverse | GGCAAAACATCTCTTGATCCTAAAGGT |
| TPP2 Canonical Probe | CTGCGCCCAGTGAGTG |
| TPP2 Alternative Forward | CTCCCTGCATAACTTTGAAGAACTG |
| TPP2 Alternative Reverse | CCTAAAGGTTTTGTTTTTGCACTCACT |
| TPP2 Alternative Probe | CACTGCGTTTCCTCATTAC |
| GCC2 Canonical Forward | GCGTTTATTGTAGAGCTTCGG |
| GCC2 Canonical Reverse | GTGGATTTTGTGCTTCTCTGATGTT |
| GCC2 Canonical Probe | TAAAATACAGCTGGCTG |
| GCC2 Alternative Forward | GAGCTGGAGGCAAGCCA |
| GCC2 Alternative Reverse | TGATGTTATTTCAGCCAGCTGTATCTG |
| GCC2 Alternative Probe | CAAGTAGAAGTCTATAAAATTTAC |
| MAP3K7 Canonical Forward | AGTGTGTCTTGTGATGGAATATGCT |
| MAP3K7 Canonical Reverse | GCAGCAGTATAATATGGCAATGGTT |
| MAP3K7 Canonical Probe | CCATGCAGCACATTAT |
| MAP3K7 Alternative Forward | TCTTGTGATGGAATATGCTGAAGGG |
| MAP3K7 Alternative Reverse | GCAGCAGTATAATATGGCAATGGTT |
| MAP3K7 Alternative Probe | AAAGAAAGGCACAAACATT |

**Table S8 Sequences of RNA pulldown**

| **Name** | **Sequence** |
| --- | --- |
| 3’Biotin_NC probe | ACCTCGGAATCTAAGACACA |
| 3’Biotin_circATP9B probe | ACACAAATCCCTGATGCAAC |

**Table S9 Sequence of FISH.**

| **Name** | **Sequence** |
| --- | --- |
| 5’Biotin_circATP9B probe | GTAACAGCCAAGACAAATCCCTGAGCAACAATGGTGCTT |

**Table S10 Sequence of siRNAs.**

| **Name** | **Sequence** |
| --- | --- |
| siNC | UUCUCCGAACGUGUCACGUTT |
| sicircATP9B#1 | TTGTTGCATCAGGGATTTG |
| sicircATP9B#2 | TGTTGCATCAGGGATTTGT |
| sicircATP9B#3 | GCATCAGGGATTTGTCTTG |
| siMYH9#1 | CCGAUAAGUAUCUCUAUGUTT |
| siMYH9#2 | GAUCAAUCCAUCUUGUGCATT |
| siMYH9#3 | CGUGCGUGCUCAUGAUAAATT |
| siMYO1A | GGUGCAUAAAGCCCAAUGATT |
| siMYO1B | GGAGGAGUAAUAAGUAACUTT |
