# Supplementary material for: Cancer-associated SF3B1 mutation suppresses DNA repair by disrupting the organization of nuclear actin network
Source: Cell Death Dis. 2026 Mar 21;17(1):334. doi: 10.1038/s41419-026-08569-5 (PMC13039298; doi:10.1038/s41419-026-08569-5)

Uncropped Western blots for Figure 2C

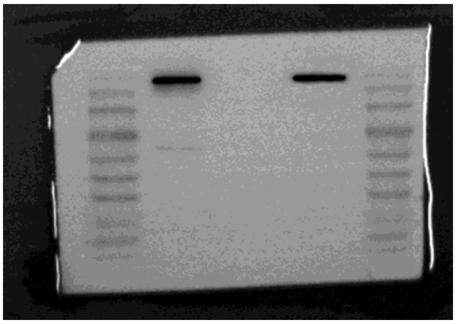

Uncropped Western blots for Figure 2H

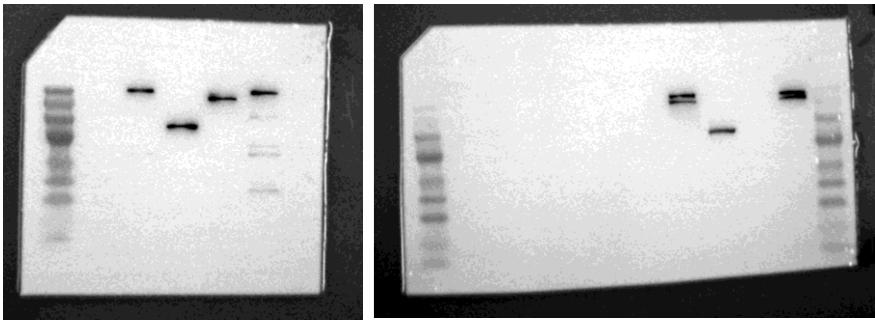

Uncropped Western blots for Figure 2J

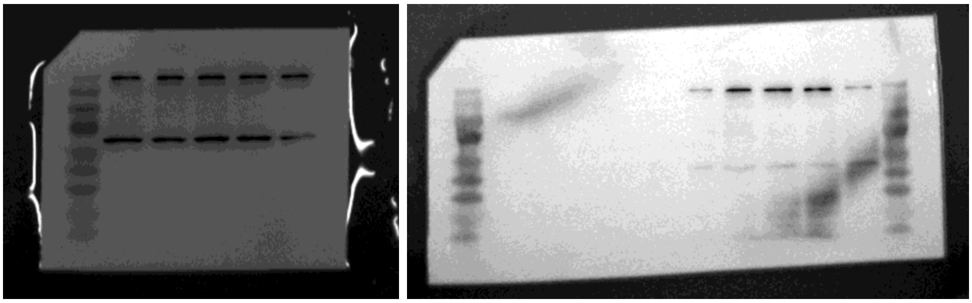

Uncropped Western blots for Figure 2L

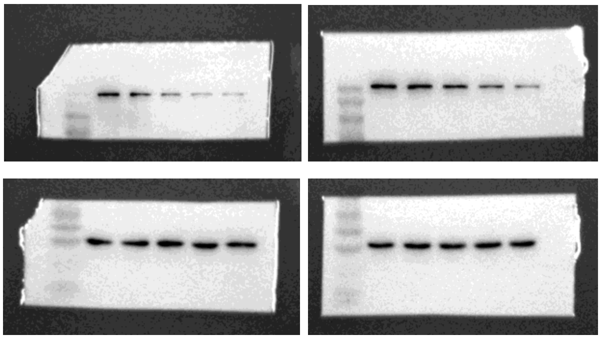

Uncropped Western blots for Figure 2N

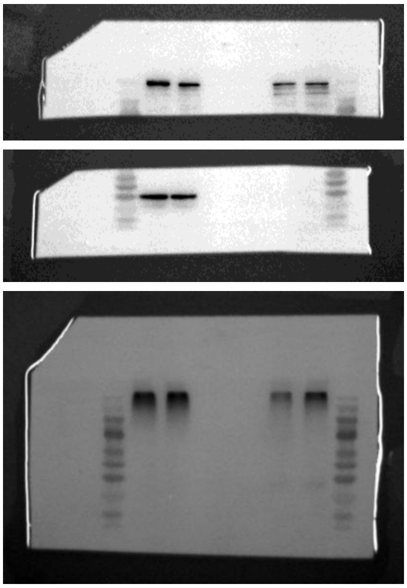

Uncropped Western blots for Figure 3F

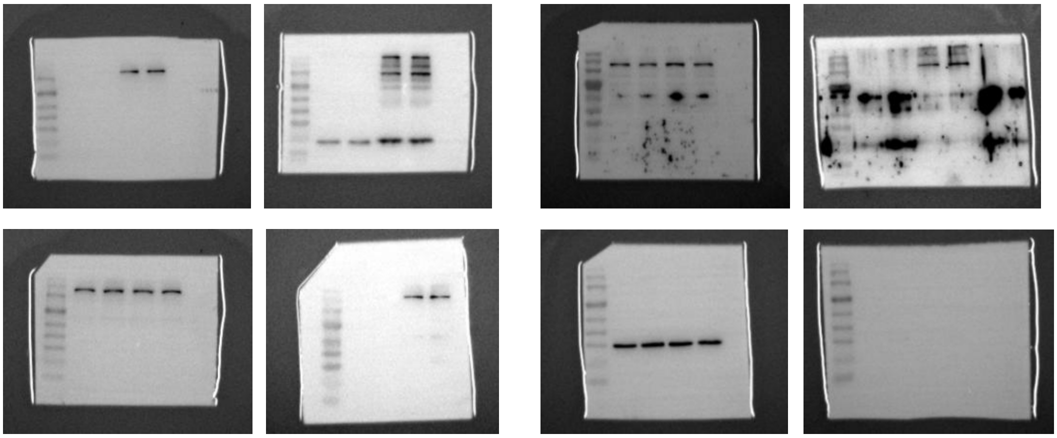

Uncropped Western blots for Figure S1C

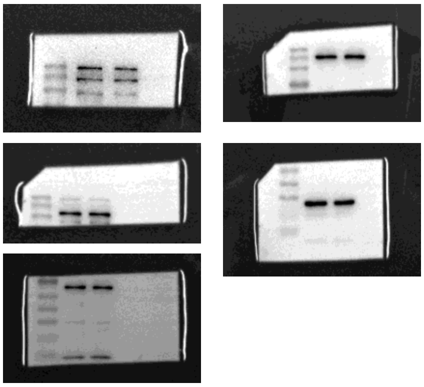

Uncropped Western blots for Figure S1D

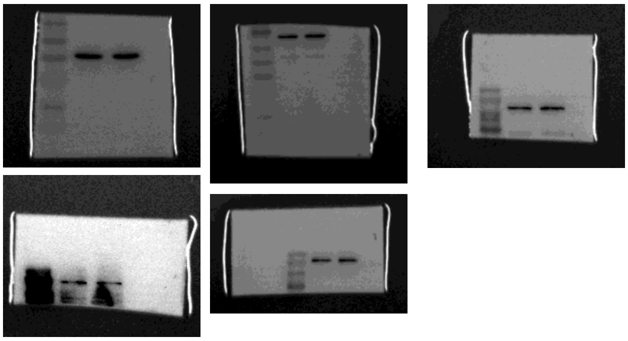

Uncropped Western blots for Figure S3C

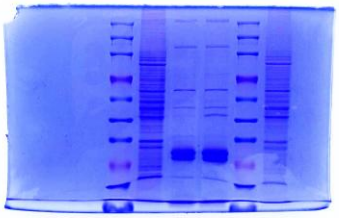

Uncropped Western blots for Figure S3D

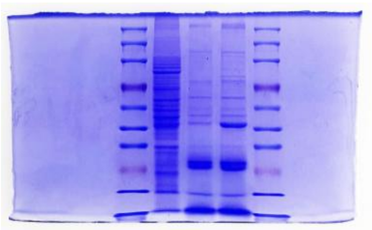

Uncropped Western blots for Figure S3F

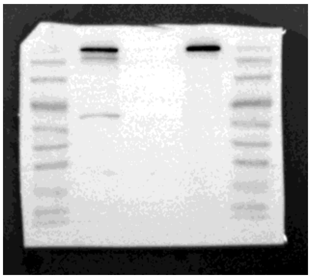

Uncropped Western blots for Figure S3I

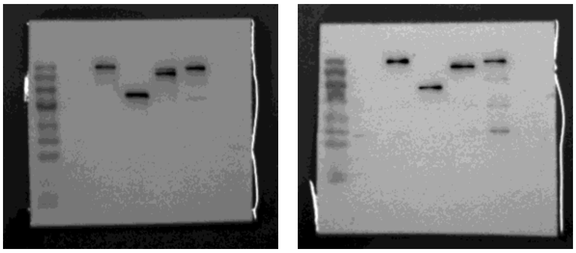

Uncropped Western blots for Figure S3Q

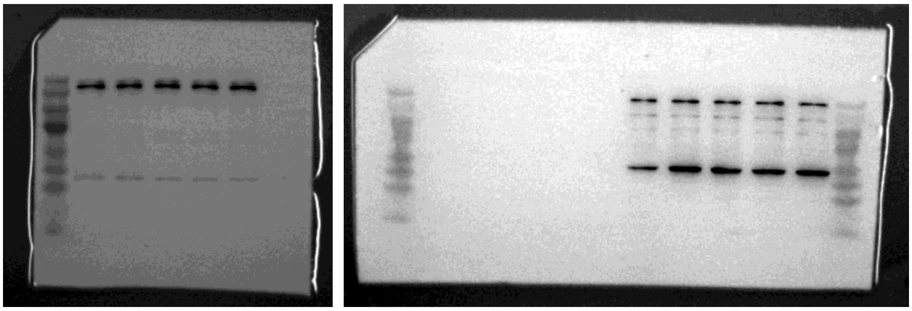

Uncropped Western blots for Figure S3V

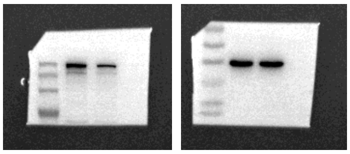

Uncropped Western blots for Figure S3W

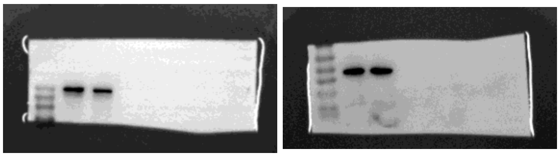

Uncropped Western blots for Figure S3X

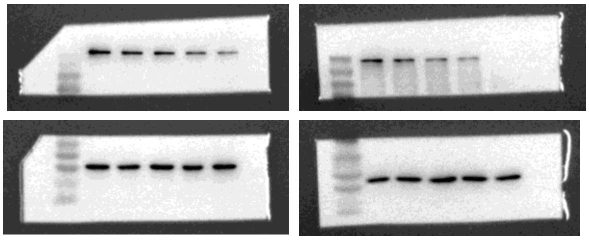

Uncropped Western blots for Figure S3Z

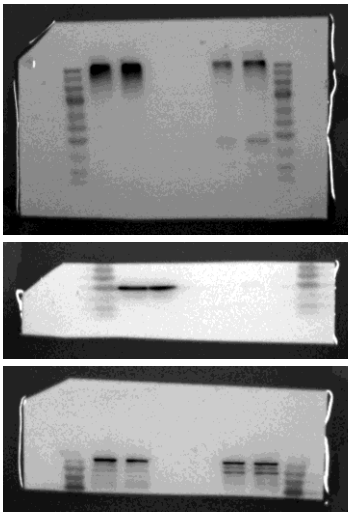

Uncropped Western blots for Figure S4D

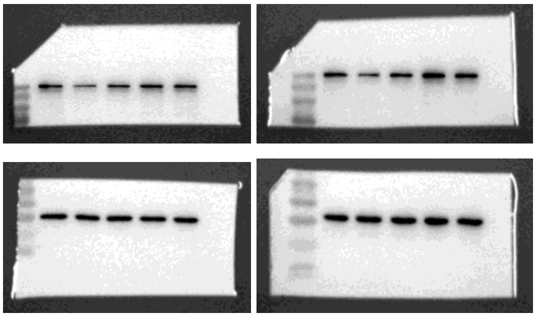

Uncropped Western blots for Figure S4E

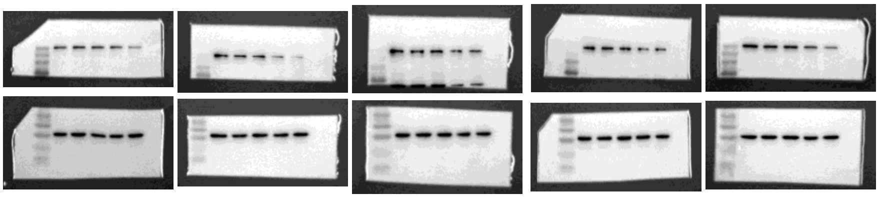

Uncropped Western blots for Figure S4F

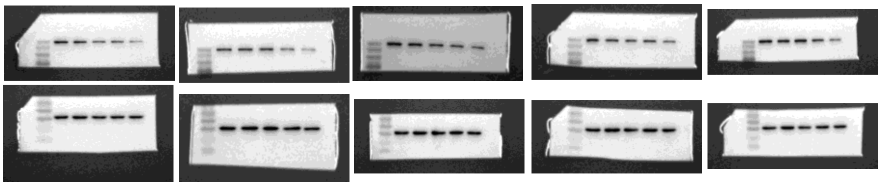

Uncropped Western blots for Figure S4I

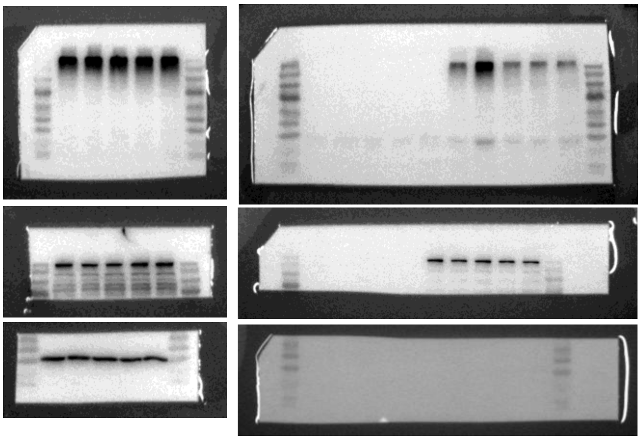

Uncropped Western blots for Figure S4J

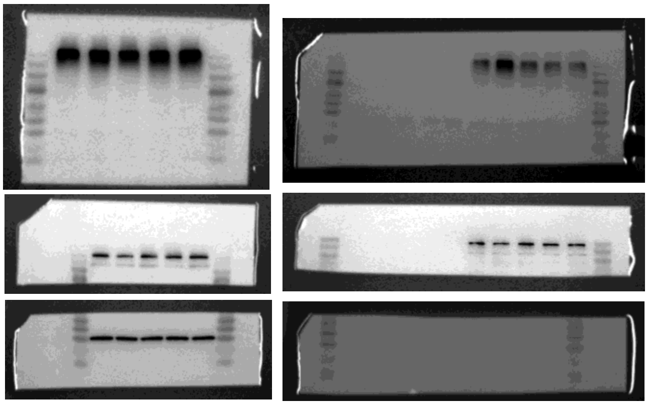

Uncropped Western blots for Figure S5B

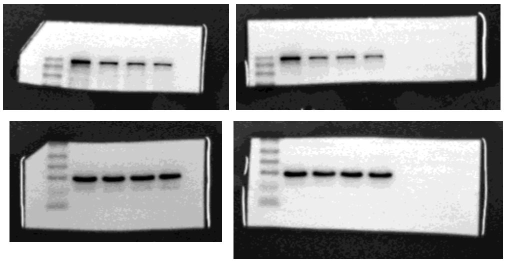

Uncropped Western blots for Figure S5G

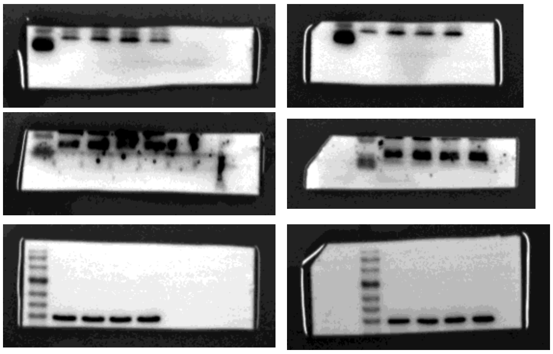

Uncropped Western blots for Figure S6D

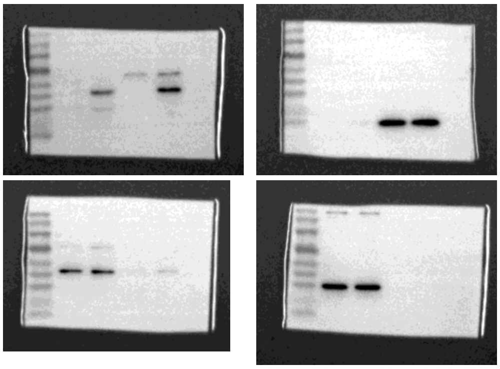

Uncropped Western blots for Figure S6G

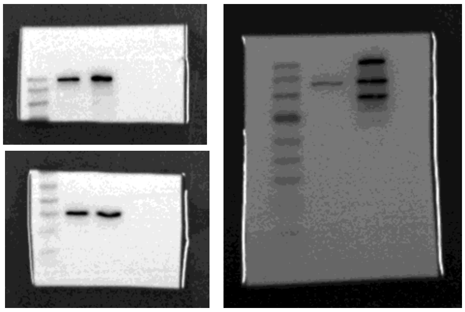

Uncropped Western blots for Figure S8A

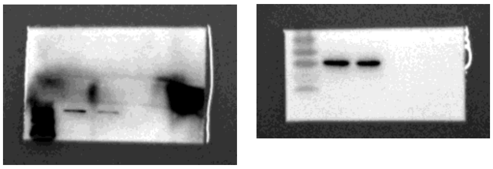

Uncropped Western blots for Figure S8B

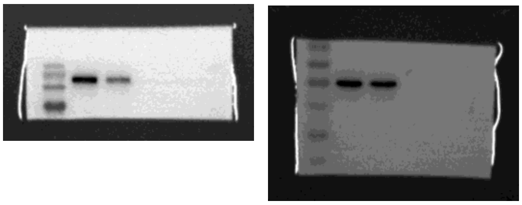

Uncropped Western blots for Figure S11C

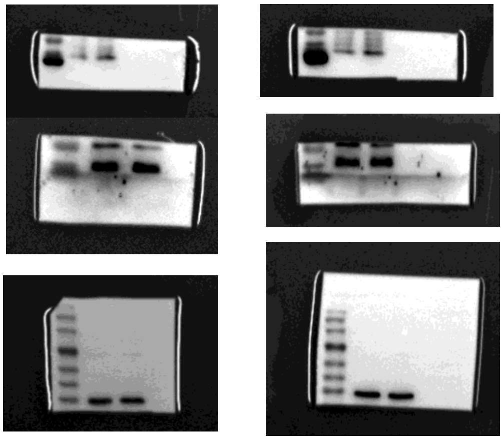

Uncropped Western blots for Figure S11F

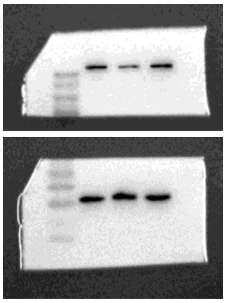

Supplement: Supplementary file 7 — Original western blots [file 41419_2026_8569_MOESM7_ESM.pdf]
